# Endogenous and Exogenous Small RNA Signatures as Novel Tools for Postmortem Interval Determination

**DOI:** 10.3390/biom16030474

**Published:** 2026-03-22

**Authors:** Yafei Wang, Botao Li, Yue Wang, Qinmin Chen, Zhonghua Wang, Guangping Fu, Shujin Li, Chenyu Zhang, Zhen Zhou, Bin Cong

**Affiliations:** 1 Hebei Key Laboratory of Forensic Medicine, Collaborative Innovation Center of Forensic Medical Molecular Identification, College of Forensic Medicine, Hebei Medical University, Shijiazhuang 050017, China; 20191051@stu.hebmu.edu.cn (Y.W.); 20211046@stu.hebmu.edu.cn (Y.W.); 23031100052@stu.hebmu.edu.cn (Q.C.); 20201055@stu.hebmu.edu.cn (Z.W.); guangpingfu@hebmu.edu.cn (G.F.); shujinli@hebmu.edu.cn (S.L.); 2 Nanjing Drum Tower Hospital Center of Molecular Diagnostic and Therapy, State Key Laboratory of Pharmaceutical Biotechnology, Jiangsu Engineering Research Center for MicroRNA Biology and Biotechnology, NJU Advanced Institute of Life Sciences (NAILS), School of Life Sciences, Nanjing University, Nanjing 210023, China; 211505009@smail.nju.edu.cn (B.L.); cyzhang@nju.edu.cn (C.Z.); 3 Research Unit of Extracellular RNA, Chinese Academy of Medical Sciences, Nanjing 210023, China; 4 Tianfu Jincheng Laboratory, Chengdu 610093, China

**Keywords:** PMI estimation, small non-coding RNAs (sncRNAs), exogenous bacterial-derived small RNAs (sRNAs), RNA degradation, PANDORA-seq, machine learning, biomarkers

## Abstract

Background: Accurate estimation of the postmortem interval (PMI), the time elapsed between death and body discovery, is a critical challenge in forensic science due to the complex interplay of factors affecting decomposition. Traditional methods based on macroscopic changes often lack precision, especially in later postmortem stages. Methods: This study aimed to develop a novel PMI estimation framework by integrating the dynamics of endogenous small non-coding RNAs (sncRNAs) and exogenous bacterial-derived small RNAs (sRNAs) using sRNA transcriptomics and machine learning. Results: Cardiac RNA degradation strongly correlated with PMI, with a random forest (RF) model achieving high accuracy (coefficient of determination (R^2^) = 0.939, mean absolute error (MAE) = 2.987 h). Employing PANDORA-seq, we profiled temporal changes in sncRNAs (miRNAs, tsRNAs and piRNAs) in postmortem cardiac tissue within 30 h in a mouse model, while simultaneously assessing RNA integrity (RIN) across eight organs. PANDORA-seq revealed stable sncRNA landscapes with specific dynamic shifts, leading to the identification of seven novel biomarkers (four tsRNAs, three piRNAs) for PMI prediction (R^2^ = 0.760, MAE = 158.990 min). Bacterial-derived sRNAs, predominantly from *Staphylococcus aureus*, were upregulated at 30 h postmortem, suggesting complementary biomarker potential. Bioinformatics analysis indicated that host miRNAs may target bacterial mRNAs, hinting at cross-kingdom interactions. Conclusion: These findings highlight the potential of integrated endogenous and exogenous sRNA analysis in PMI estimation, providing a high-precision, rapid diagnostic tool and revealing complex postmortem molecular processes.

## 1. Introduction

The postmortem interval, defined as the elapsed time between death and body discovery, represents a critical parameter in forensic science. Accurate estimation of PMI is essential for criminal investigations, forensic identification, and biomedical research [1,2]. However, due to the influence of multiple endogenous and exogenous factors—such as environmental conditions, individual variability, and decomposition stage—PMI estimation remains a major challenge in forensic practice [2,3,4]. Traditional approaches primarily rely on macroscopic postmortem changes (e.g., algor mortis, rigor mortis and livor mortis) or environmental analyses, but their accuracy is limited, particularly during the intermediate and late postmortem stages [5,6,7].

Recent advances in molecular biology and microbiology have provided innovative perspectives for PMI estimation [8]. Decomposition is a complex biological process influenced by microbial colonization and environmental factors [9]. The dynamic succession of microbial communities has emerged as a promising dimension for PMI estimation. Accumulating evidence indicates that bacterial communities undergo time-dependent, predictable shifts during decomposition [10], potentially functioning as a “biological clock” [11]. For instance, Zhang et al. demonstrated that RF models trained on microbial profiles from soil, skin and rectal samples of murine cadavers achieved an R^2^ of 86.83% and an MAE of only 1.82 days within 60 days postmortem, with Actinobacteria, Proteobacteria and Firmicutes serving as the most robust predictors. Advances in high-throughput sequencing, including 16S rRNA gene sequencing, have significantly improved the taxonomic resolution of bacterial communities [10]. Furthermore, sequencing of eukaryotic 18S rRNA and fungal internal transcribed spacer regions has provided complementary insights into PMI estimation. Belk et al. similarly constructed accurate predictive models of PMI using 16S rRNA gene markers [12].

In parallel with microbial studies, PMI inference based on biomolecular degradation has also received considerable attention [13]. Nucleic acids (DNA and RNA) have become research hotspots due to their time-dependent degradation patterns [7]. Although the degradation rate is influenced by environmental temperature [14], tissue type [15], and individual factors [16,17], resulting in considerable variability, these degradation rules still provide a crucial foundation for PMI estimation.

In RNA research, small non-coding RNAs (sncRNAs) have attracted particular interest due to their stability and biological functions. Among them, microRNAs (miRNAs) have been widely applied in PMI inference [18]. miRNAs, typically consisting of 20–24 nucleotides, regulate gene expression by binding to target mRNAs and may play active roles in postmortem biological processes such as apoptosis [19,20]. Multiple studies have revealed postmortem expression dynamics of miRNAs in different tissues (e.g., heart, brain, liver, skeletal muscle) and body fluids (e.g., vitreous humor, blood) [18]. For instance, in brain tissue, Ma et al. found that miR-9 and miR-125b remain stable for up to 144 h under 10–35 °C conditions, suggesting their suitability as endogenous reference molecules [21]. Similarly, Lv et al. reported that in human cadaver samples, cardiac miR-1 and miR-133a remain stable for more than five days postmortem, whereas liver-specific miR-122 showes a marked decline with increasing PMI, especially under high-temperature conditions, highlighting significant inter-organ variability in miRNA stability [22].

Taken together, this study systematically investigated the postmortem dynamics of diverse sncRNA classes to identify critical biomarkers and establish a high-accuracy model for PMI estimation. Using PANDORA-seq, we characterized the temporal expression patterns of sncRNAs—including tsRNAs, piRNAs and miRNAs—in cardiac tissue within 30 h after death and revealed distinct postmortem expression signatures of cardiac miRNAs [23]. We further integrated PANDORA-seq data with PCR array validation and machine learning approaches, leading to the identification of seven novel PMI-associated biomarkers (tsRNAs and piRNAs) and the development of a rapid and highly accurate prediction model. In addition, we preliminarily explored the potential contribution of microbe-derived sRNAs and the interplay between host miRNAs and microbial RNAs in postmortem processes.

## 2. Materials and Methods

### 2.1. Experimental Animals

This study utilized 149 male, C57BL/6J, 10–12 weeks old mice (Beijing HFK Bioscience Co., Ltd., Beijing, China). All experimental procedures were approved by the Laboratory Animal Ethical and Welfare Committee of Hebei Medical University, with the animal ethics review approval number: IACUC-Hebmu-2021010. After sacrificed by cervical dislocation, the mice were placed in a supine position in a constant temperature and humidity chamber (LHS-HC-I series, Shanghai Yiheng Scientific Instrument Co., Ltd., Shanghai, China) under experimental conditions of 25 degrees Celsius and 50% humidity [24]. After rinsing with physiological saline, all samples were soaked overnight in RNAlater Stabilization Solution (Cat. No. AM7021, Thermo Fisher Scientific, Waltham, MA, USA) at −4 °C. After removal, they were transferred to a freezer and stored at −80 °C until use. All samples were ground for RNA extraction.

### 2.2. Experimental Methods

#### 2.2.1. RIN Analysis

RIN testing was performed on 48 mice at postmortem time points of 0 h, 6 h, 12 h, 18 h, 24 h, 30 h, 36 h, and 48 h (*n* = 6) in a constant-temperature and humidity chamber. Eight types of tissues and organs were obtained, including lung, liver, muscle, brain, heart, testis, spleen and kidney.

According to the manufacturer’s instructions, after frozen grinding of 30 mg–50 mg of tissue, RNA was extracted with 700 μL Trizol reagent (Cat. No. 15596026), Thermo Scientific, Waltham, MA, USA). The Qubit RNA HS assay kit (Cat. No. Q32852, Thermo, Scientific, Waltham, MA, USA ) was used to quantify the concentration of 2 μL of extracted RNA. The sample was diluted to about 100 ng/μL according to the RNA concentration, and the RIN was checked on the Agilent 4200 fragment analyzer system (Cat. No. G2991AA, Agilent Technologies, Santa Clara, CA, USA).

#### 2.2.2. PANDORA-Seq Profiling

PANDORA-seq samples were obtained from 30 mice at post-mortem time points of 0, 6, 12, 18, 24 and 30 h (*n* = 5).

Total RNA was extracted using 1mL TRIzol Reagent. sRNAs of 15–50 nucleotides were selected.

##### Treatment of RNA with AlkB and T4PNK

The Escherichia coli AlkB gene was cloned into the NdeI/BamHI restriction sites of the pET28a(+) plasmid to enable expression of the AlkB protein with a hexahistidine tag at the N-terminus. The resulting plasmid was transformed into the *E. coli* BL21(DE3) strain for protein expression. The transformed *E. coli* cells were cultured in lysogeny broth (LB) medium supplemented with 50 µg/mL kanamycin. Protein expression was induced by adding 1 mM isopropyl β-d-1-thiogalactopyranoside (IPTG), and the culture was incubated at 37 °C for 3 h. The AlkB protein was purified using an Ni-NTA Superflow column. After purification, the protein was stored at −80 °C in a buffer containing 20 mM Tris-HCl (pH 8.0), 50% glycerol, 0.2 M NaCl, and 2 mM dithiothreitol (DTT). The purity of the AlkB protein was assessed by 12% SDS-PAGE.

The RNA was incubated in a 50 μL reaction mixture containing 50 mM HEPES (pH 8.0), 75 μM ferrous ammonium sulfate (pH 5.0), 1 mM α-ketoglutaric acid (Cat. No. K1128-25G, Sigma-Aldrich, St. Louis, Mo, USA ), 2 mM sodium ascorbate, 50 mg L^−1^ bovine serum albumin, 4 μg mL^−1^ AlkB (Guangzhou Epibiotek Co., Ltd., Guangzhou, China), 2000 U RNase inhibitor and 200 ng RNA at 37 °C for 30 min. After incubation at 37 °C for 30 min, the mixture was purified with TRIzol reagent. Then, 5 μL of 10× PNK buffer (New England Biolabs, ipswich, MA, USA), 5 μL of 1 mM ATP (New England Biolabs, Ipswich, MA, USA), and 1 μL of T4PNK (New England Biolabs, Ipswich, MA, USA) were added, and the mixture was incubated at 37 °C for 20 min. The mixture was subsequently purified with TRIzol reagent and then subjected to sRNA sequencing.

##### Small RNA Library Construction and Deep Sequencing

The RNA segment was separated by PAGE; then a 15–45 nucleotide strip was selected and recycled. The adapters were obtained from the QIAseq^®^ miRNA Library Kit (Cat. No. 331505, Qiagen, Hilden, Germany) and ligated sequentially. The amplified flow cell was sequenced on the Illumina system by epibiotek (Guangzhou Epibiotek Co., Ltd., Guangzhou, China).

##### Small RNA Annotation and Analysis for PANDORA-Seq Data

Annotation of sRNA sequences was performed using SPORTS1.1 (an updated version of SPORTS1.0), with a tolerance for one mismatch (SPORTS1.1 parameter setting: −M 1) [25]. All RNA reads were mapped to the following databases: the rRNA and YRNA databases from the National Center for Biotechnology Information: https://www.ncbi.nlm.nih.gov/nuccore (accessed on 6 December 2023); the miRNA database miRbase 21: https://www.mirbase.org/ (accessed on 6 December 2023); the piRNA databases from piRBase: http://bigdata.ibp.ac.cn/piRBase/ (accessed on 6 December 2023) and piRNABank: accessed on 6 December 2023; the tRNA databases from GtRNAdb: http://gtrnadb.ucsc.edu/ (accessed on 6 December 2023) and MINT map: https://cm.jefferson.edu/mintmap/ (accessed on 6 December 2023); non-coding RNAs were annotated by Ensembl: http://www.ensembl.org/index.html (accessed on 6 December 2023) and Rfam 12.3: http://rfam.xfam.org/ (accessed on 6 December 2023). Using the procedures outlined below, mature tRNA sequences were extracted from the sequence datasets of GtRNAdb and mitotRNAdb: (1) predicted introns were removed; (2) a CCA sequence was added to the 3′ ends of all tRNAs; (3) a G nucleotide was added to the 5′ end of histidine tRNAs. The tsRNAs were categorized into four types based on the origin of the tRNA loci: 5′ tsRNA (derived from the 5′ end of pre-/mature tRNA); 3′ tsRNA (derived from the 3′ end of pre-tRNA); 3′ tsRNA-CCA end (derived from the 3′ end of mature tRNA); and internal tsRNAs (not derived from 3′ or 5′ loci of tRNA). To achieve unique annotation of each rsRNA, sRNAs were mapped to their corresponding parent rRNAs following an ascending order based on rRNA sequence length during rsRNA annotation. (For instance, rsRNAs mapped to 5.8S rRNA would not be further aligned to the genomic region where 5.8S and 45S rRNAs overlap).

##### Sequencing Data Processing

Raw sequencing reads were preprocessed using fastp (v0.22.0) [26], which performed adapter trimming, quality filtering, and removal of low-quality reads. Alignment of endogenous and exogenous RNAs was conducted using Bowtie (v1.0.0) [27] with default parameters: -n 2 -l 28 -e 70 -q --phred33-quals -k 1 --fr -I 0 -X 250 -p 1.

For endogenous RNAs, the mouse reference genome and mRNA reference sequences were obtained from NCBI: https://www.ncbi.nlm.nih.gov/datasets/genome/GCF_000001635.27 (accessed on 7 July 2025). The genome assembly was used directly for alignment, whereas the mRNA reference dataset was extracted and filtered from the same genome assembly. For exogenous RNAs, the Staphylococcus aureus reference genome was downloaded from NCBI: https://ftp.ncbi.nlm.nih.gov/genomes/all/GCF/000/013/425/GCF_000013425.1_ASM1342v1 (accessed on 7 July 2025).

Annotation of endogenous sRNAs was performed using SPORTS1.1 [28] with default settings for 15–45 nucleotides. Exogenous sRNAs were defined as sequencing reads that did not map to the mouse reference genome.

##### miRNA Temporal Clustering

Dynamic expression patterns of miRNAs were analyzed using Fuzzy C-Means (FCM) clustering implemented in the Mfuzz R package (v2.62.0) [29]. miRNA expression values were first standardized according to the standard Mfuzz workflow, and FCM clustering was applied to identify groups of miRNAs exhibiting similar postmortem temporal expression profiles.

##### miRNA Target Prediction and Functional Enrichment

Predicted mouse mRNA targets for miRNAs were obtained from the TargetScanMouse database (Release 8.0) using the “Predicted Targets context++ scores” file [30]. To improve reliability, miRNAs with average read counts ≤ 5 were excluded, and target genes with context++ scores > −0.2 or a percentile < 90 were filtered out. Gene Ontology (GO) enrichment analysis of the filtered target genes was performed in Python (v3.9.21) using the gseapy package (v1.1.4).

##### Differential Expression Analysis of Exogenous sRNAs

Differential expression analysis of exogenous sRNAs was conducted using pydeseq2 (v0.4.12) in Python (v3.9.21). Prior to analysis, low-abundance fragments with total read counts < 1000 were excluded. Fragments were considered significantly differentially expressed if they exhibited a fold change > 2 and a q-value < 0.01.

##### Taxonomic Assignment of Exogenous sRNAs

The species origin of exogenous sRNAs was determined via NCBI BLAST: https://blast.ncbi.nlm.nih.gov/Blast.cgi (accessed on 8 July 2025), using BLAST with the search set restricted to Bacteria (taxid: 2), while the other parameters were set to default. Only hits with 100% query coverage, 100% sequence identity, and an E-value < 10^−5^ were retained for downstream analysis.

##### Prediction of Staphylococcus aureus Targets by Mouse miRNAs

To explore potential cross-kingdom regulation, candidate mRNA targets were selected from the CDS regions of the *Staphylococcus aureus* reference genome (NCBI, GCF_000013425.1_ASM1342v1). miRNAs with average read counts ≤ 5 were excluded to ensure data robustness. Target prediction was performed using RNAhybrid (v2.1.2) [31] with parameters -b 3 -e -30 -f 2,8 -u 4 -v 4 -d nothing -m 100000.

#### 2.2.3. PCR Array Validation

For the PCR array, heart tissue was obtained at a random time point 1470 min after the death of 71 mice. Commercially available quantitative real-time PCR arrays were used to analyze and screen the expression profiles of 20 target genes related to RNA molecules. This study used the PCR chip detection method and strictly followed the manufacturer’s instructions (Wcgene Biotech, Shanghai, China). After extracting total RNA from the myocardium, cDNA was synthesized by reverse transcription and was finally analyzed using a PCR chip. All data were analyzed and processed using software provided by Wcgene Biotech [32]. The ΔCt calculation method represents the relative RNA expression level [33], which is normalized to GAPDH. All primer sequences are provided in the Supplementary Materials.

### 2.3. Statistical Analysis and Data Visualization

All preprocessing, filtering, and statistical analyses of tabular data were performed in Python (v3.9.21) using the NumPy (v1.24.3) and pandas (v2.2.3) libraries. Data visualization was carried out using matplotlib (v3.9.4) and seaborn (v0.13.2), with the exception of FCM clustering, which was conducted separately.

Normalized expression levels were quantified as reads per million mapped reads (RPM) and used for downstream analyses, including differential expression assessment and FCM clustering. Statistical significance of differences between each postmortem time point and 0 h was evaluated using Student’s *t*-test, followed by false discovery rate (FDR) correction using the Benjamini–Hochberg procedure. Significance thresholds were set as follows: FDR-adjusted *p* < 0.05 (*), FDR-adjusted *p* < 0.01 (**), and FDR-adjusted *p* < 0.001 (***).

All machine learning analyses were performed in Python (v3.9.21) using the scikit-learn (v1.6.0) [34], xgboost (v2.1.4) [35] and shap (v0.48.0) [36] packages.

#### 2.3.1. RIN-Based Machine Learning Models

For regression modeling of RIN data, four algorithms with distinct characteristics were evaluated:

(1) LASSO regression [37], which employs L1 regularization for feature selection; (2) Elastic Net regression [38,39], combining L1 and L2 regularization; both linear models were optimized using 10-fold cross-validation to select regularization parameters; (3) RF regression [40] with 100 decision trees, leveraging ensemble learning to reduce overfitting; (4) Artificial Neural Networks (ANNs) with a double-hidden-layer architecture (50–20 neurons), ReLU activation, and the Adam optimizer.

Model performance was evaluated using 10-fold cross-validation and three metrics: R^2^, MAE, and root mean squared error (RMSE). Feature contributions were assessed using standardized absolute coefficients for linear models, Gini importance for random forests, and the sum of absolute input-to-hidden-layer weights for ANNs. SHapley Additive exPlanations (SHAP) analysis was conducted using LinearExplainer, TreeExplainer or KernelExplainer to interpret model predictions and assess the contribution of individual organ RIN values to PMI estimation.

#### 2.3.2. PANDORA-Seq-Based Feature Selection and Modeling

For PANDORA-seq data, the data with zero standard deviation were removed through mfuzz analysis. Recursive feature elimination combined with random forests was then applied, employing repeated three-times five-fold cross-validation over 40 random seeds. For each seed, an optimal feature subset was determined, yielding 40 subsets in total. Features present in >35 subsets were selected as candidate biomarkers. Models were implemented in R (4.3.2). After the primers were designed, RNA with poor specificity was removed to determine the RNA molecules for the final model. The ΔCt calculation method represents the relative RNA expression level, which is normalized to GAPDH.

#### 2.3.3. PCR Array-Based Machine Learning Models

PCR Array data were used to evaluate nine regression algorithms: RF (500 trees, sqrt feature selection), K-Nearest Neighbors (3 neighbors, distance weighting), two XGBoost variants (linear and DART boosters), Support Vector Regression (radial basis and linear kernels), Ridge regression (leave-one-out CV), LASSO regression (leave-one-out CV), and ANN (50–20 hidden layers). Fifteen-fold cross-validation was applied to validate all models. Model performance was assessed using R^2^, MAE and RMSE, and results were visualized using three-dimensional scatter plots for comprehensive comparison.

#### 2.3.4. RF Recursive Feature Elimination and Feature Importance

To optimize model efficiency and reduce feature redundancy, a nested cross-validation framework was implemented using a random forest (RF) regressor with recursive feature elimination and cross-validation (RFECV). The RF regressor was set with 500 trees, square-root feature selection, min_samples_split = 3, and min_samples_leaf = 1. A 5-fold outer cross-validation was used for performance evaluation, while RFECV was conducted within each training fold in the inner 5-fold cross-validation to avoid information leakage, with R^2^ as the evaluation metric. Model stability was further assessed through multi-seed testing across 990 random seeds (10–999), and the top 40 seeds ranked by R^2^ were retained for downstream analysis. Features selected in at least two inner folds within each seed were collected, and the most frequently recurring sncRNA features across these optimal seeds were identified as core biomarkers. The final RF model was constructed using these core features with identical hyperparameters and validated using 15-fold cross-validation to ensure robust and unbiased estimation of predictive performance, with R^2^, MAE and RMSE reported as evaluation metrics.

Feature contributions were evaluated using both Gini importance and SHAP analysis. SHAP values were computed using TreeExplainer and visualized as beeswarm plots, illustrating the direction and magnitude of each feature’s impact, and as bar plots, showing the mean absolute SHAP values to reflect overall contribution. Features were color-coded by biological category (piRNA or tsRNA) to facilitate interpretation.

#### 2.3.5. Linear Regression Analysis

For comparative assessment, linear regression models were constructed to examine the relationship between organ RIN values or PCR Array biomarker expression and postmortem time. Models were implemented in Python (v3.9.21) using scipy (v1.13.1). Regression lines were plotted in red, with corresponding regression equations, R^2^ and *p*-values annotated on the figures.

## 3. Results

### 3.1. Cardiac RNA Degradation Estimates PMI Effectively

**Figure 1:**
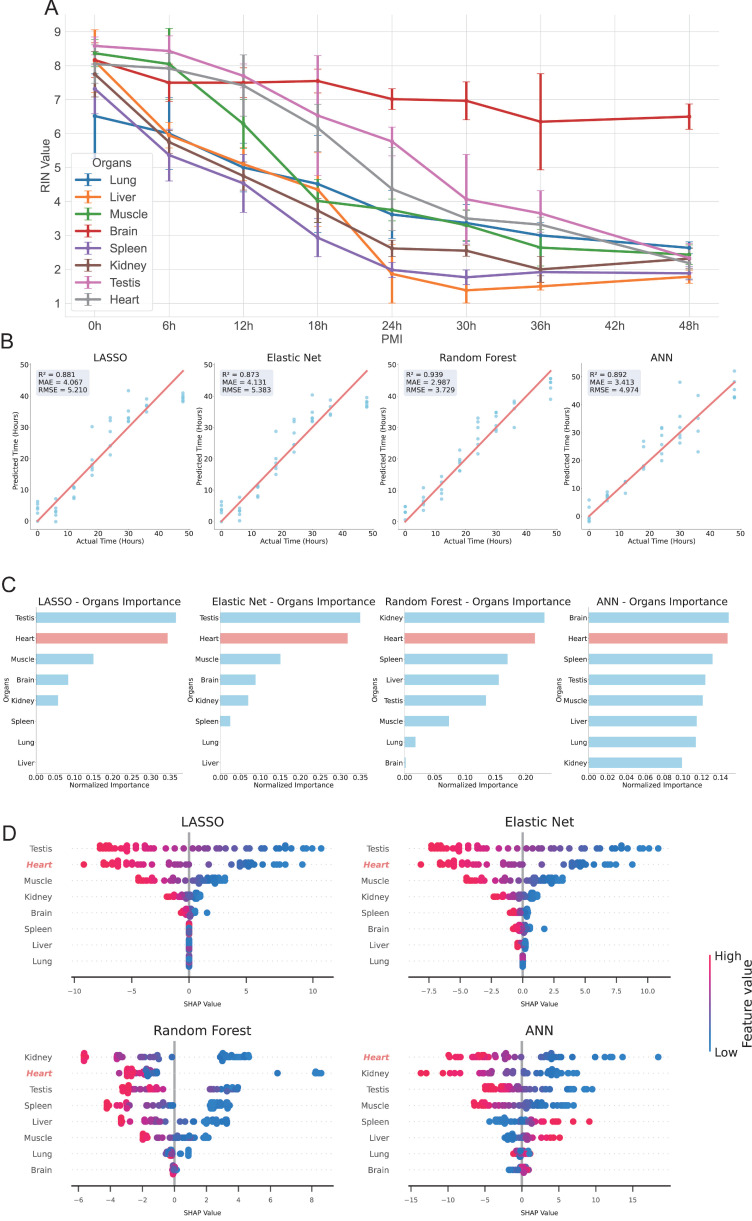
RIN Evaluation in Eight Mouse Organs and PMI Estimation within 48 Hours Post-Decapitation. (**A**) Average RIN values (*n* = 6) and standard deviations across eight organs at different postmortem time points. The line graph shows the trend of mean RIN values over time, with error bars indicating standard deviations at each time point. (**B**) Comparison of estimation performance for four regression models (LASSO, Elastic Net, RF, ANN) in predicting PMI (hours). Scatter plots show predicted vs. actual values, with the red diagonal line representing ideal estimation. Evaluation metrics include R^2^, MAE (hours) and RMSE (hours). (**C**) Importance ranking of organ features for PMI estimation across models. Normalized importance scores (sum = 1) highlight the heart (red) as the most important feature across all models, with other organs shown in blue. (**D**) SHAP value analysis of feature contributions for PMI estimation in LASSO, Elastic Net, RF, and ANN models. The heart feature is shown in red, with standardized feature values visualized as “High-Low”.

Postmortem RNA degradation occurs over time, and several studies have utilized RNA degradation characteristics to estimate the PMI [41,42]. The RIN Number is a key indicator used to assess RNA quality [43,44], and it has been applied to characterize RNA degradation. We assessed the RIN values of RNA extracted from eight organs or tissues—lung, liver, muscle, brain, heart, testis, spleen and kidney—at postmortem time points of 0 h, 6 h, 12 h, 18 h, 24 h, 30 h, 36 h and 48 h (*n* = 6) after decapitation (Figure 1, Supplementary Figure S1). Correlation analysis revealed that RIN values were negatively correlated with PMI across all eight organs. Notably, brain tissue exhibited the slowest rate of integrity decline, while the remaining organs showed no significant differences in degradation rates (Figure 1A).

To determine which organ’s RIN values were most strongly correlated with PMI, we constructed time-dependent linear regression models for the RIN values of each of the eight organs. Among the regression models, the heart model exhibited the highest coefficient of determination (R^2^), indicating the best fit. The testis, muscle, spleen and kidney models also showed relatively strong fits, while the lung, liver and brain models displayed weaker fits (Supplementary Figure S1). These results suggest that RNA degradation in the postmortem heart may exhibit more pronounced temporal and regular patterns, which renders the heart an ideal organ for combining RIN studies with PMI estimation.

To further enhance the estimation of PMI using RIN values, we constructed four machine learning models—Lasso regression, Elastic Net regression, RF and ANN—using the RIN values from the eight organs as input features to predict the time of death. Cross-validation results indicated that the predicted values were highly consistent with the actual observations (Figure 1B). Specifically, the estimation performances of the four models were as follows: Lasso regression (R^2^ = 0.881, MAE = 4.067 h, RMSE = 5.210 h), Elastic Net regression (R^2^ = 0.873, MAE = 4.131 h, RMSE = 5.383 h), RF (R^2^ = 0.939, MAE = 2.987 h, RMSE = 3.729 h), and ANN (R^2^ = 0.892, MAE = 3.413 h, RMSE = 4.974 h). These results demonstrate that RNA degradation features from multiple organs can effectively predict PMI.

We then performed feature importance analysis to further identify the most important organ contributing to the model. The results showed that the heart RIN values were significantly important across all four models (ranked second overall) (Figure 1C), suggesting a strong correlation between cardiac RIN and PMI. For a more comprehensive evaluation of the contributions of each organ, we conducted SHAP analysis. SHAP results (Figure 1D) indicated that the heart feature had the second-largest SHAP value distribution in the Lasso regression, Elastic Net regression and RF models, while in the ANN model, the heart feature showed the broadest SHAP value distribution. These consistent findings highlight that, among the eight organs, the RNA degradation characteristics of the heart make the most significant contribution to PMI estimation.

Together, these results indicate that cardiac RIN is strongly correlated with PMI, and that, among the organs studied, the heart is the most advantageous for RNA degradation-based PMI estimation (with testis showing sex-specific differences).

### 3.2. Dynamic Changes in Cardiac sncRNA Expression Within 30 Hours Postmortem

While cardiac RIN is strongly correlated with PMI, the RIN Number predominantly reflects rRNA quality [43], rendering it insensitive to the degradation of mRNAs and sncRNAs. To comprehensively assess RIN, we employed PANDORA-seq [23] (Panoramic RNA Display by Overcoming RNA Modification Aborted Sequencing) to profile cardiac tissue at postmortem time points of 0, 6, 12, 18, 24 and 30 h. PANDORA-seq is an advanced sRNA sequencing method that overcomes the limitations of conventional approaches, thereby enabling the discovery of previously undetected sncRNAs, including tsRNAs and rsRNAs. This technique has also been applied to construct comprehensive sRNA landscapes in human body fluids and animal plasma [25].

**Figure 2:**
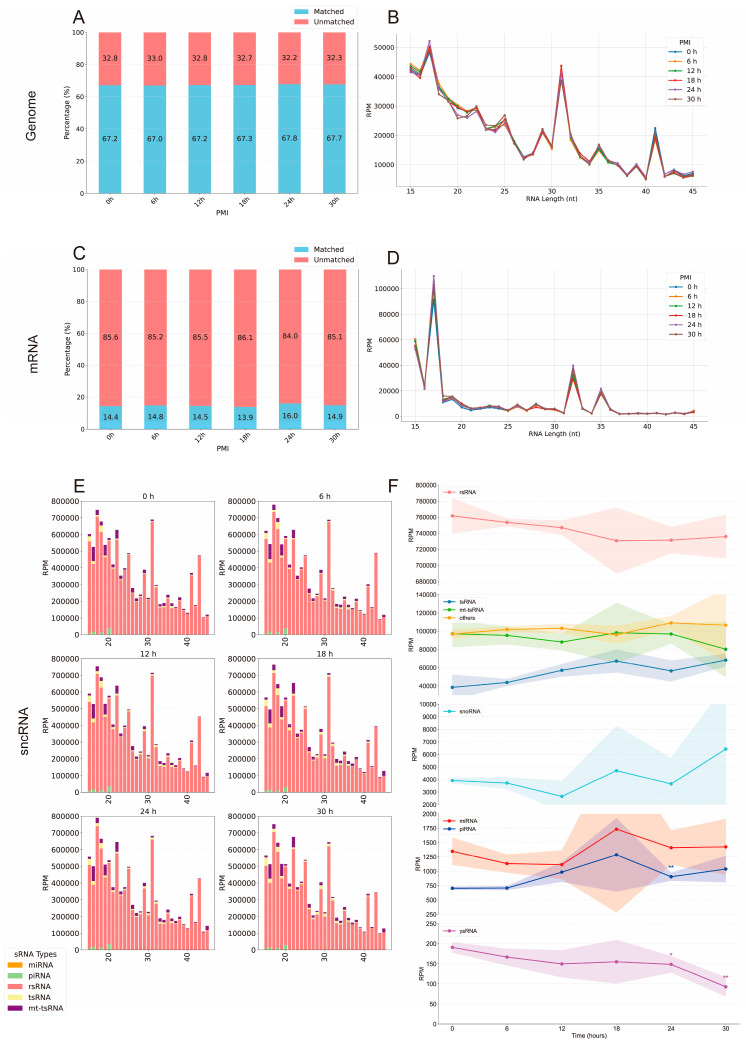
Dynamic Small RNA Profiling in Cardiac Tissue within 30 Hours Postmortem by PANDORA-seq. (**A**) Proportion of sRNAs mapped to the mouse reference genome (average, *n* = 5). (**B**) Distribution of sRNA fragment lengths mapped to the mouse reference genome (average, *n* = 5). (**C**) Proportion of mRNA fragments mapped to the mouse mRNA reference sequence (average, *n* = 5). (**D**) Distribution of mRNA fragment lengths mapped to the mouse mRNA reference sequence (average, *n* = 5). (**E**) Length distribution of five sRNA types at six time points (average, *n* = 5). (**F**) Expression trends of various sRNAs over time (RPM, Reads Per Million), normalized. * *p* < 0.05; ** *p* < 0.01, comparing data to 0 h (*t*-test with Benjamini–Hochberg FDR correction). The *x*-axis represents time (hours) and the *y*-axis shows RPM (mean ± SD, *n* = 5), with shaded areas indicating standard deviation.

Mapping analyses revealed that the proportion of sRNAs aligned to the mouse genome remained stable across all time points (Figure 2A), with the length distribution of mapped sRNAs showing no significant variation (Figure 2B), indicating that the total abundance and size profile of endogenous sRNAs in cardiac tissue are largely preserved within 30 h postmortem. Similarly, mRNA-derived fragments mapped to the reference genome exhibited no significant changes in abundance or fragment length distribution (Figure 2C,D), suggesting that cardiac mRNAs largely remain intact and do not undergo substantial fragmentation into 15–45 nt fragments during this period.

To investigate the postmortem dynamics of various sncRNAs, we analyzed their fragment lengths and read counts. The length distributions of all five major sncRNA classes remained largely unchanged throughout the 30 h period (Figure 2E), indicating minimal degradation. However, total RPM analysis revealed pronounced temporal changes in specific sncRNA classes (Figure 2F). tsRNAs exhibited a marked accumulation over time, with total RPM increasing from ~38,000 at 0 h to ~57,000 at 12 h (50% increase) and further rising to ~67,000 at 18 h (76% cumulative increase), maintaining elevated levels at 24 h and 30 h. piRNAs also displayed upregulation between 12 h and 30 h, with RPM increasing from <750 to ~1000, whereas ysRNAs showed a significant decline, decreasing by approximately 50% at 30 h relative to 0 h. Other sncRNA classes displayed variable temporal patterns, with rsRNAs remaining relatively stable. Overall, rsRNAs, mitochondrial tsRNAs (mt-tsRNAs), and tsRNAs were the most abundant sncRNA classes, while piRNAs, snoRNAs, miRNAs, and other sncRNAs exhibited RPM values below 10,000.

Collectively, these results demonstrate that the overall abundance and length distributions of endogenous sRNAs, including sncRNAs, are preserved in postmortem cardiac tissue up to 30 h. In contrast, the expression levels of specific sncRNA species exhibit distinct temporal dynamics, providing a multidimensional molecular framework for PMI estimation. Specifically, tsRNA accumulation may serve as a reliable marker for the early postmortem phase (0–18 h), whereas changes in piRNA and ysRNA levels provide complementary information during the 12–30 h window. Importantly, several sncRNAs—including tsRNAs and piRNAs—show increasing expression over time, challenging the conventional view that RNA uniformly degrades postmortem. These findings suggest the persistence of regulated molecular processes in the postmortem heart, potentially reflecting residual biological activity. Of course, simpler explanations such as differential stability, RNA fragmentation patterns, changes in sequencing/ligation efficiency, or PANDORA-seq chemical bias cannot be ruled out. Further research is warranted.

### 3.3. Temporal Clustering Analysis Reveals Dynamic Expression Patterns of Postmortem Cardiac miRNAs

Although the global dynamics of sncRNA classes offer valuable insights into postmortem molecular changes, microRNAs (miRNAs) are among the most well-characterized regulators of gene expression, functioning through mRNA cleavage or translational repression (MicroRNAs: genomics, biogenesis, mechanism and function). Given their established roles in cellular regulation, we focused on the temporal expression patterns of miRNAs to gain deeper mechanistic insights into postmortem cardiac metabolism.

**Figure 3:**
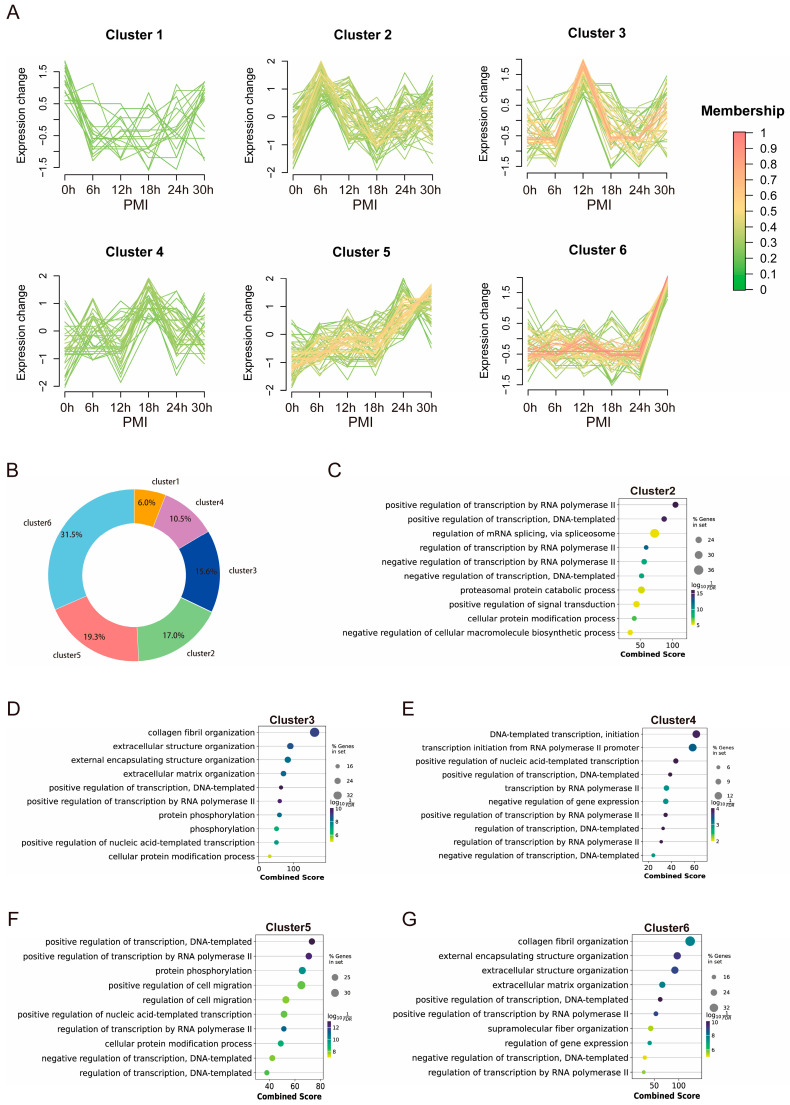
Dynamic Analysis of miRNA Expression in Postmortem Mouse Cardiac Tissue. (**A**) Fuzzy membership values for miRNAs assigned to six clusters (Cluster 1–6) at six postmortem time points (0 h, 6 h, 12 h, 18 h, 24 h, 30 h). Rows represent individual miRNAs, and columns represent PMI time points. Color intensity indicates the degree of membership (scale: 0 to 1). Each cluster peaks at different PMI time points. (**B**) Relative abundance of miRNAs within each cluster as a percentage of the total miRNAs across all clusters. (**C**–**G**) Gene Ontology (GO) enrichment analysis of target genes for miRNAs in (**C**) Cluster 2 (peak at 6 h), (**D**) Cluster 3 (peak at 12 h), (**E**) Cluster 4 (peak at 18 h), (**F**) Cluster 5 (high at 24–30 h), and (**G**) Cluster 6 (peak at 30 h). Each plot shows the enriched biological processes for the corresponding miRNA cluster.

To elucidate the temporal expression patterns of different miRNAs, we applied the FCM clustering algorithm to classify miRNAs at six postmortem time points into six distinct clusters (Cluster 1–6) (Figure 3A). Each cluster exhibited peak expression at different time points. In terms of the number of miRNAs, Cluster 6 contained the largest number, followed by Cluster 5, Cluster 2, Cluster 3 and Cluster 4, with Cluster 1 containing the fewest miRNAs (Figure 3B).

To further elucidate the potential regulatory functions of each cluster, we predicted the target mRNAs of miRNAs within Clusters 2–6 and performed GO enrichment analysis on these predicted targets. The analysis revealed that the target genes of each cluster were significantly enriched in specific biological pathways, highlighting distinct temporal and functional expression patterns (Figure 3C–G).

Cluster 2 miRNAs peaked at 6 h postmortem. The GO enrichment analysis of its target genes was the most complex compared to the other clusters, with hallmark pathways including regulation of mRNA splicing via spliceosome, negative regulation of transcription by RNA polymerase II, proteasomal protein catabolic process, positive regulation of signal transduction, and negative regulation of cellular macromolecule biosynthetic processes. These pathways are broadly involved in gene expression regulation, the balance between synthesis and degradation, and signal integration. This suggests that, around 6 h postmortem, the heart tissue might produce a significant amount of Cluster 2 miRNAs, which could inhibit their target genes, triggering complex downstream cascades.

Cluster 3 miRNAs peaked at 12 h postmortem, and Cluster 6 miRNAs showed significant upregulation at 30 h. The target genes of these two clusters were mainly involved in extracellular matrix (ECM) formation and tissue structuring, including collagen fiber organization and the formation of various extracellular structures. Representative pathways included collagen fibril organization, extracellular structure organization, and external encapsulating structure organization. These findings suggest that between 12 and 30 h postmortem, the heart tissue may secrete specific miRNAs (Cluster 3 and Cluster 6) to regulate ECM-related gene expression, which affects the structural integrity of cardiac tissue.

Cluster 4 miRNAs peaked at 18 h postmortem, and Cluster 5 miRNAs were elevated between 24 and 30 h. The target genes of these two clusters were primarily related to transcription regulation, including both positive and negative regulation (e.g., positive regulation of transcription, DNA-templated; positive regulation of transcription by RNA polymerase II). We speculate that between 18 and 30 h postmortem, the heart secretes a large amount of related miRNAs that, through complex regulatory mechanisms, modulate transcription processes and affect downstream metabolic events in cardiomyocytes. Additionally, the target genes of Cluster 5 were significantly enriched in pathways related to protein phosphorylation and cell migration (e.g., protein phosphorylation; positive regulation of cell migration; regulation of cell migration), suggesting that after 24 h postmortem, significant changes in cardiomyocyte and heart tissue structure may occur, with miRNAs playing an important regulatory role in this process.

### 3.4. Multiple tsRNAs and piRNAs in Cardiac Tissue as Biomarkers for PMI Estimation

**Figure 4:**
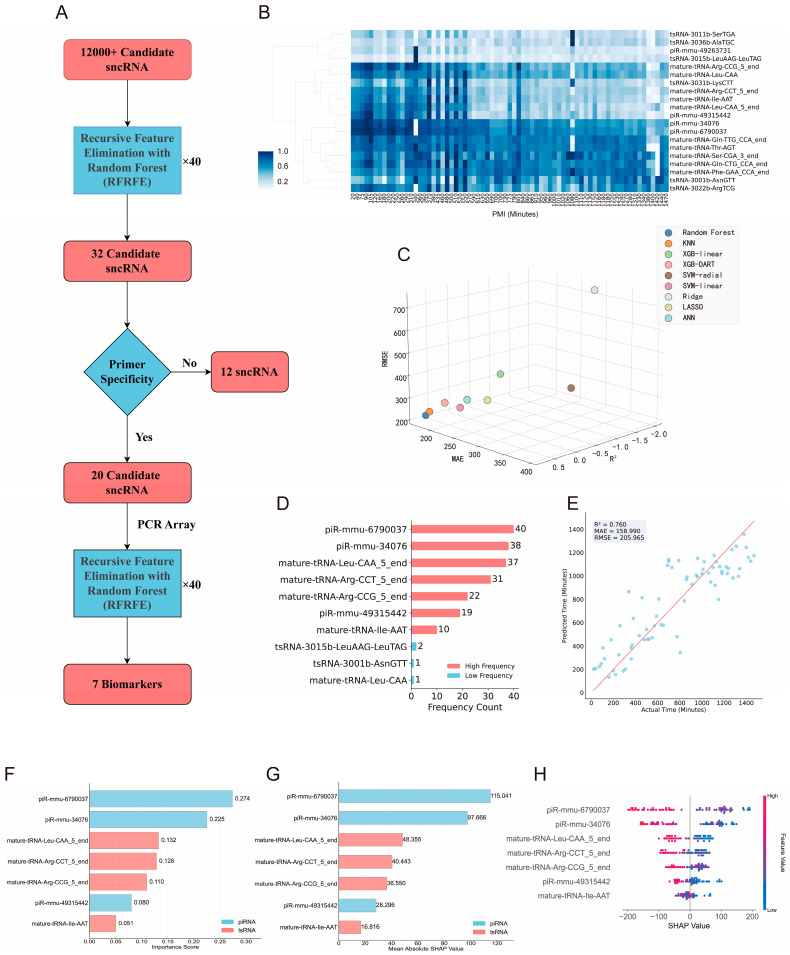
Biomarker Screening and Model Construction for PMI Estimation Based on PANDORA-seq, PCR Array, and Machine Learning. (**A**) Workflow for selecting biomarkers to efficiently predict PMI. Red boxes represent candidate sncRNAs or biomarkers, and blue boxes represent screening technologies. (**B**) PCR Array results for 20 candidate sncRNAs. The *x*-axis represents PMI (in minutes), and color intensity indicates expression abundance (Z-score normalized). (**C**) Comparison of performance across nine machine learning models using PCR Array data from 20 sncRNAs. The three axes represent the model’s R^2^, MAE and RMSE (MAE and RMSE in minutes). (**D**) Final selection of seven key biomarkers (4 tsRNAs and 3 piRNAs, marked in red) identified using recursive feature elimination within a nested cross-validation framework and 15-fold cross-validation. (**E**) Performance evaluation of the final RF model constructed using the seven biomarkers, with the red diagonal line representing ideal predictions. The upper left corner shows evaluation metrics, with MAE and RMSE in minutes. (**F**) Importance score ranking of the seven biomarkers in the final RF model. (**G**) Feature importance ranking (Mean Absolute SHAP Value) for the seven biomarkers in the final model. (**H**) Beeswarm plots showing the impact of each biomarker on the final model. SHAP values quantify the direction and magnitude of each biomarker’s effect on the model’s output, with positive values indicating extended predicted time and negative values indicating the opposite. “High-Low” represents feature value size.

Given the significant dynamic expression patterns of sncRNAs, we explored whether changes in the expression of specific sncRNAs could more accurately estimate PMI. To achieve this, we integrated PANDORA-seq, PCR Array technology and machine learning models to systematically analyze the relationship between sncRNA expression changes in postmortem cardiac tissue and PMI (Figure 4A).

Initially, using sequencing data, we employed a three-repeat five-fold cross-validation combined with recursive feature elimination in random forests, iterating over 40 random seeds. We selected 32 candidate sncRNAs based on frequency and type, including 26 tsRNAs and 6 piRNAs (Figure 4A). To construct a rapid detection system and improve estimation accuracy, we screened 20 sncRNAs (16 tsRNAs and 4 piRNAs) based on primer specificity for PCR Array validation with shorter inter-group time intervals. The results revealed significant temporal expression patterns for several molecules (Figure 4B).

Subsequently, using the PCR Array data for these 20 sncRNAs, we built nine machine learning models, with the RF model showing the best performance in cross-validation, yielding good estimation results: R^2^ = 0.726, MAE = 171.782 min, RMSE = 220.034 min (Figure 4C, Supplementary Figure S2). To further accelerate detection speed and enhance model estimation power, we applied recursive feature elimination within a nested cross-validation framework and validated models using 15-fold cross-validation, ultimately identifying seven biomarkers (4 tsRNAs and 3 piRNAs). The expression levels of these biomarkers were all significantly negatively correlated with time (Supplementary Figure S3), and the RF model constructed with these biomarkers showed a significant improvement in estimation accuracy (R^2^ = 0.760, MAE = 158.990 min, RMSE = 205.965 min). Although the sncRNA-based model showed a lower coefficient of determination (R^2^ = 0.760) compared to the multi-organ RIN model (R^2^ = 0.939), it achieved a smaller MAE (158.990 min vs. 179.220 min) and lower RMSE (205.965 vs. 223.740 min), indicating superior estimation accuracy in absolute time within the first 30 h postmortem. Importance analysis (Figure 4F–H) revealed that piR-mmu-6790037 and piR-mmu-34076 were the most critical contributors to the model. For the top six biomarkers, higher expression levels were associated with lower PMI estimations (Figure 4H), suggesting a negative correlation between their abundance and PMI.

The final predictive model, which relies solely on PCR Array quantification of four tsRNAs and three piRNAs, provides an efficient approach for PMI estimation. Overall, tsRNAs and piRNAs exhibited an increasing expression trend in the heart over the postmortem period (Figure 2F). Monitoring the expression levels of these specific tsRNAs and piRNAs enables precise PMI estimation. These results imply that tsRNAs and piRNAs may retain functional significance in postmortem cardiac tissue, potentially reflecting residual metabolic activity up to 30 h after death.

### 3.5. Bacterial-Derived sRNAs as Potential Biomarkers for PMI Estimation

Endogenous sncRNAs in postmortem cardiac tissue displayed temporally dynamic expression patterns and demonstrated strong predictive power for PMI estimation. This observation prompted us to examine whether exogenous, bacterial-derived sRNAs also exhibit time-dependent changes.

**Figure 5:**
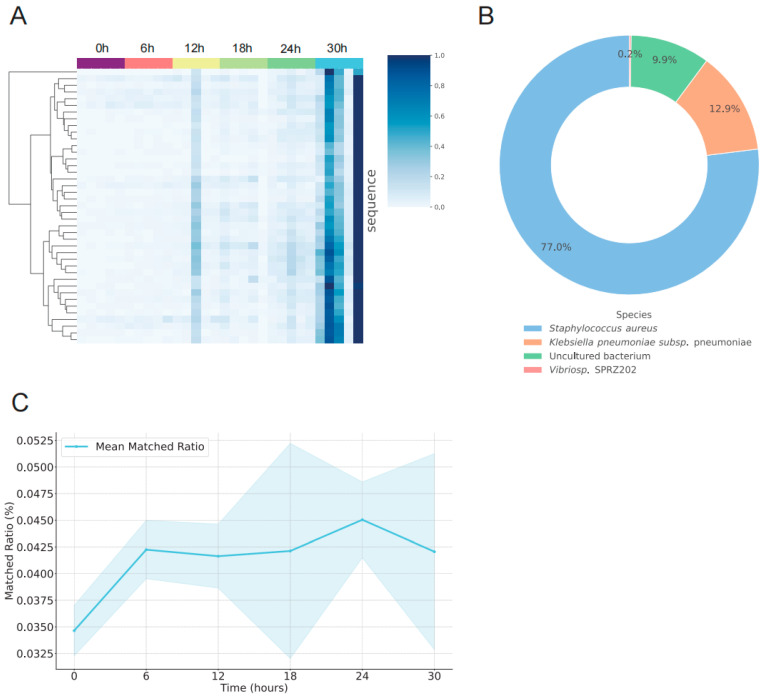
Expression Characteristics of Microbial-derived sRNAs at Postmortem Time Points. (**A**) Heatmap showing read counts for 41 microbial-specific differential fragments, with red indicating high expression and blue indicating low expression. Data were Z-score normalized to eliminate sequence-specific differences. (**B**) Species composition of the 41 microbial-specific differential fragments. (**C**) Line plot illustrating changes in the matching rate between sRNAs and the Staphylococcus aureus genome from 0 to 30 h postmortem (mean ± SD, *n* = 5). *X*-axis: postmortem time (h); *Y*-axis: matching rate (%). The blue line denotes the mean and the light-blue shading represents the standard deviation range.

Using differential expression analysis combined with NCBI BLAST alignment, we identified 41 distinct microbe-specific sRNA fragments from sequencing reads that did not map to the mouse reference genome. These fragments were markedly upregulated at 30 h postmortem (Figure 5A), a pattern absent at earlier time points, suggesting their potential as biomarkers for estimating a PMI of approximately 30 h.

Taxonomic profiling indicated that, under conditions of 25 °C and 50% humidity, these microbial fragments predominantly originated from *Staphylococcus aureus* (77%), followed by *Klebsiella pneumoniae* subsp. (12.9%), uncultured bacteria (9.9%) and *Vibrio* sp. *SPRZ202* (0.1%) (Figure 5B). Temporal profiling of *S. aureus*-derived fragments revealed a gradual increase from 0.036% at 0 h to 0.042% at 6 h (a 17% rise), peaking at 0.045% at 24 h, with no significant changes thereafter (Figure 5C). These findings indicate that, although *S. aureus*-specific fragments were strongly detected at 30 h postmortem, their overall abundance did not substantially increase, implying that the observed sRNA dynamics likely reflect alterations in microbial community composition or metabolic activity rather than simple bacterial proliferation.

Collectively, these data demonstrate that bacterial-derived sRNAs—particularly in the 24–30 h postmortem window—hold promise as biomarkers for PMI estimation. Integrating both endogenous sncRNA and exogenous sRNA dynamics may enhance predictive accuracy.

### 3.6. Potential Regulation of Staphylococcus aureus Metabolism by Endogenous miRNAs

The pronounced increase in *Staphylococcus aureus*-specific fragments at 30 h postmortem, without a corresponding substantial rise in total bacterial abundance, suggests that this upregulation may reflect alterations in microbial community structure or metabolic states rather than an expansion of bacterial load.

**Figure 6:**
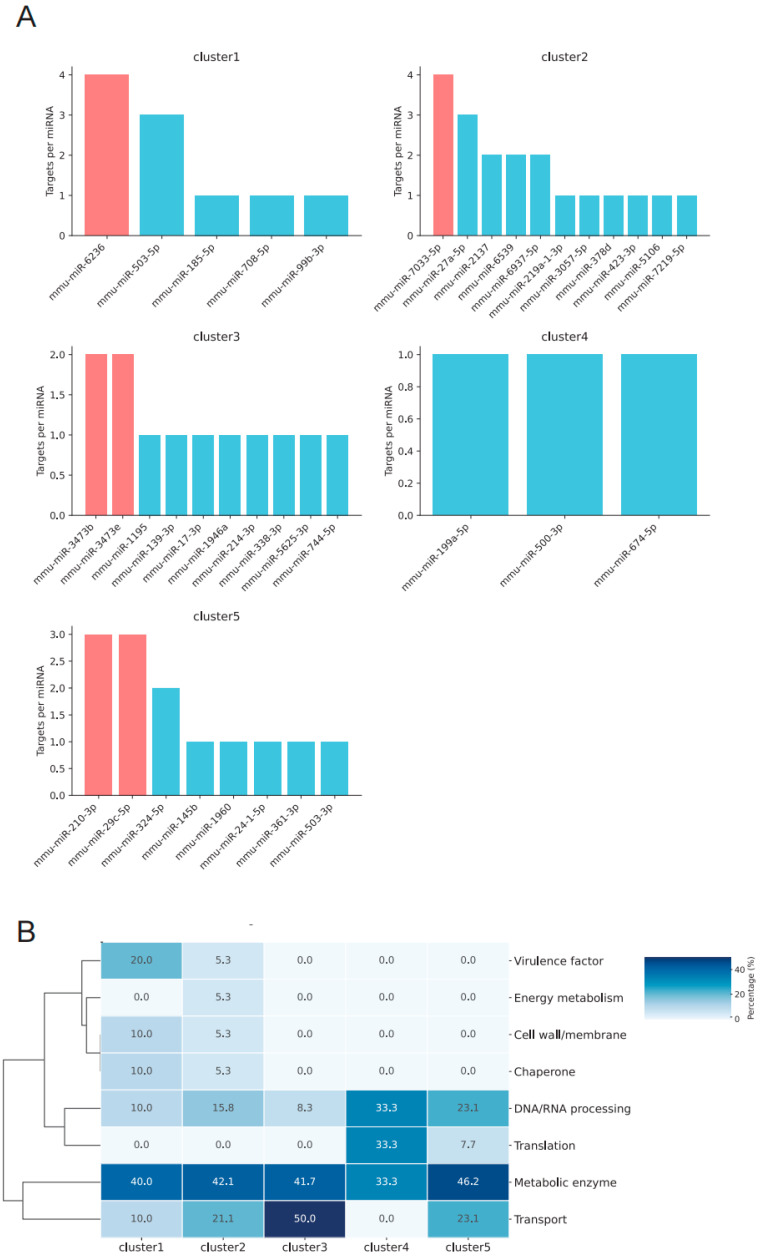
Endogenous Cardiac miRNAs Targeting *Staphylococcus aureus* mRNAs Postmortem. (**A**) Bar plots showing the number of predicted *Staphylococcus aureus* mRNA targets for highly expressed miRNAs in Clusters 1–5. In each subplot, the miRNA with the highest target count is highlighted in red, while the others are shown in blue. (**B**) Heatmap illustrating the predicted binding of miRNAs to *S. aureus* mRNA targets. Color intensity reflects the proportion of target counts, with darker shades indicating higher values. Each cell is annotated with its corresponding percentage.

Given that endogenous cardiac miRNAs display temporally dynamic expression postmortem (Figure 3A), we next investigated their potential role in modulating microbial metabolism. Analysis of highly expressed miRNAs from Clusters 1–5 revealed that multiple members within each cluster were predicted to target *S. aureus* transcripts, suggesting potential cooperative regulatory effects (Figure 6A). Functional categorization of these predicted targets uncovered two major features: (1) a shared targeting signature across all clusters, with significant enrichment for mRNAs encoding metabolic enzymes; and (2) cluster-specific preferences, whereby Cluster 1 miRNAs predominantly targeted virulence factor-encoding mRNAs, whereas Clusters 4 and 5 miRNAs preferentially targeted mRNAs involved in DNA/RNA processing and translation (Figure 6B).

Based on the above bioinformatics analysis results, we propose an innovative hypothesis: Potential Regulation of *Staphylococcus aureus* Metabolism by Endogenous miRNAs. This indicates that even 30 h after death, myocardial tissue may still retain residual metabolic activity and biological responsiveness.

## 4. Discussion

Our study successfully developed a novel framework for PMI estimation by integrating endogenous and exogenous sRNA transcriptomic data with machine learning techniques. By moving beyond traditional analytical perspectives, we systematically explored the dynamic changes in postmortem rRNA, mRNA and various sncRNAs (including miRNA, tsRNA, piRNA, rsRNA and ysRNA), as well as bacterial-derived sRNAs.

This integrative approach not only reveals the complex and orderly molecular processes that occur in the heart after death, but also demonstrates that changes in RNA after death are not merely random degradation events.

A core finding of our study is the feasibility and high precision of using sncRNAs as biomarkers for PMI estimation. RIN assessments primarily reflect the degradation status of rRNA, which has certain limitations. To address this, we employed PANDORA-seq technology to deeply explore the dynamic changes in the sncRNA landscape in cardiac tissue within 30 h postmortem. Surprisingly, the overall landscape of sncRNAs did not exhibit global degradation; instead, it maintained relative stability, particularly with the temporal dynamics of specific non-coding RNA species, such as the accumulation of tsRNAs and piRNAs and the decline of ysRNAs. These regulated changes suggest the presence of active molecular processes postmortem, rather than mere passive degradation, establishing sncRNAs as a novel class of functional biomarkers for PMI estimation.

Building on these findings, we successfully identified a biomarker panel composed of seven sncRNAs (four tsRNAs and three piRNAs). This panel, combined with PANDORA-seq, PCR Array technology, and machine learning models, enabled highly accurate PMI estimation (MAE = 158.990 min). This result demonstrates the feasibility of translating omics discoveries into a qPCR-based targeted detection system suitable for forensic applications, highlighting a paradigm shift from multi-omics research to high-precision, rapid diagnostic tools. Additionally, our preliminary analysis revealed that among various organs, cardiac tissue exhibited the most consistent and predictable RNA degradation dynamics. A machine learning model based on multi-organ data (e.g., RF, R^2^ = 0.939) further underscored the strong potential of multi-feature omics approaches in enhancing estimation accuracy and robustness. This finding provides a practical guide for selecting biomarkers in forensic practice and underscores the promising applications of sncRNAs in PMI estimation.

Another key finding of this study is the extension of the multi-omics perspective beyond endogenous RNAs to explore the potential of microbial-derived sRNAs in PMI estimation. We identified bacterial sRNAs, particularly from *Staphylococcus aureus*, that were significantly upregulated approximately 30 h postmortem. As a commensal bacterium, *Staphylococcus aureus* stably colonizes the skin and nasal cavities of healthy mice under normal physiological conditions without causing apparent infection. In contrast, the heart of a healthy mammal is typically considered a sterile organ under physiological homeostasis. Our findings indicate that *Staphylococcus aureus* undergoes postmortem migration and proliferation following host death. Previous studies have validated that *S. aureus* can rapidly disseminate via the short mucosal blood heart route. Compared with intestinal bacteria such as *Escherichia coli*, *S. aureus* exhibits a shorter migration distance, fewer physiological barriers, and a higher survival rate during the early postmortem period [45]. Our findings further support this hypothesis. The discovery positions microbial sRNAs as valuable exogenous biomarkers that complement host-derived signals, especially in longer PMI windows. The inclusion of exogenous sRNAs introduces a new dimension to PMI estimation, suggesting that the dynamic succession of microbial communities and their molecular signals may be closely linked to the progression of time. Notably, since the present study is solely based on PANDORA-seq data and lacks direct experimental validation of bacterial identity, the above conclusion cannot be fully confirmed. Nevertheless, these results offer a novel candidate target for the development of biomarkers for early PMI estimation.

Previous studies have suggested that host microRNAs (miRNAs) can regulate the composition and function of the microbiome by modulating gene expression [46]. Against this backdrop, we conducted bioinformatics analyses to investigate the potential regulatory role of host endogenous miRNAs in *Staphylococcus aureus* metabolism. Based on our findings, we propose a new hypothesis: during a specific postmortem time window, the migration of *Staphylococcus aureus* from the extracellular to the intracellular environment of the host may be regulated by cardiac miRNAs derived from postmortem tissue. This suggests that even 30 h after death, cardiac muscle tissue may retain residual metabolic activity and biological responsiveness. However, the underlying regulatory mechanisms remain unclear and require further experimental validation. The integration of host transcriptomics and microbiomics provides a new perspective for future research on postmortem dynamics and time-of-death estimation.

Our study further explored the potential biological significance of the specific expression of endogenous sncRNAs and clarified that postmortem tissue is not a purely degrading environment. By applying the FCM clustering algorithm to the temporal clustering of miRNAs, we revealed distinct miRNA expression clusters, each closely associated with the coordinated regulation of specific biological pathways. These pathways span from early stress responses and gene regulation (6 h), to ECM organization (12–30 h), and finally to later transcriptional and cell migration processes (24–30 h). This structured, temporally phased regulatory network suggests that postmortem cardiac tissue may retain surprising levels of biological activity. Consistent with prior literature, this observation reinforces the notion that RNA fate following death is governed by active regulatory mechanisms, rather than being confined to passive degradation. It indicates that miRNA expression profiles not only serve as biomarkers for PMI estimation but also offer a window into the pathophysiological state of postmortem tissues.

The observed increase in the expression of certain sncRNAs further prompts consideration of whether there is sustained transcriptional activity or selective stability mechanisms postmortem. The active RNA stabilization mechanisms and temporally regulated expression observed in cardiac tissue suggest that its cellular structure and function may retain partial integrity for some time after death. This finding fundamentally expands our understanding of postmortem biological activity. The early postmortem heart is not in a chaotic state of degradation, but rather undergoes a series of complex yet orderly metabolic processes.

Nevertheless, this study has certain limitations. Firstly, the validation of PANDORA-seq and PCR Array was performed exclusively in cardiac tissue. Emerging evidence has demonstrated that the expression and degradation patterns of sncRNAs exhibit obvious tissue specificity, which may restrict the broader applicability of the identified biomarkers across different tissue types. In future work, we will perform more in-depth investigations to evaluate the specificity and universality of these biomarkers in multiple tissues. Secondly, the research on interactions between MicroRNAs and microbes proposed by bioinformatics analysis needs further experimental validation to improve accuracy.

In summary, this study demonstrates the powerful potential of multi-omics strategies in advancing the discovery of biomarkers for PMI estimation. These findings offer practical tools for forensic science. Future work will focus on validating these biomarker combinations in human samples under different environmental conditions, experimentally confirming the potential cross-regulatory mechanisms, and further exploring the potential applications of these biomarkers in forensic science.

## 5. Conclusions

These findings highlight the potential of integrated endogenous and exogenous sRNA analysis in PMI estimation, providing a high-precision, rapid diagnostic tool and revealing complex postmortem molecular processes.

## Data Availability

The original contributions presented in this study are included in the article/Supplementary Materials. Further inquiries can be directed to the corresponding authors.

## Supplementary Materials

The following supporting information can be downloaded at: https://www.mdpi.com/article/10.3390/biom16030474/s1, Figure S1: Each subpanel displays the distribution of RIN values and their linear fitting results for a specific organ (e.g., heart, liver, brain, etc.) at different postmortem intervals; Figure S2: Each subpanel presents the performance evaluation of regression models (including RF, KNN, XGB-linear, XGB-DART, SVM-radial, SVM-linear, Ridge, LASSO, and ANN) for PCR array data of 20 sncRNAs; Figure S3: Temporal expression patterns and linear fitting of seven biomarkers; Figure S4: histogram of PMI distribution of all samples. Figure S5: PCR Array reaction conditions; Table S1: Primer sequences for 20 biomarkers.

## Abbreviations

Abbreviation	Full Name
PMI	Postmortem Interval
sncRNA	Small Non-coding RNA
sRNA	Small RNA
RF	Random Forest
R^2^	Coefficient of Determination
MAE	Mean Absolute Error
PANDORA-seq	PANDORA sequencing
miRNA	MicroRNA
tsRNA	Transfer RNA-derived small RNA
piRNA	PIWI-interacting RNA
RIN	RNA integrity
mRNA	Messenger RNA
rsRNA	Ribosomal RNA-derived small RNA
FCM	Fuzzy C-Means
GO	Gene Ontology
RPM	Reads per million mapped reads
ANN	Artificial Neural Network
RMSE	Root Mean Square Error
SHAP	SHapley Additive exPlanations
RFECV	Recursive Feature Elimination with Cross-Validation
ysRNA	Y RNA-derived small RNA
ECM	Extracellular Matrix
