# Supplementary material for: Endogenous and Exogenous Small RNA Signatures as Novel Tools for Postmortem Interval Determination
Source: Biomolecules. 2026 Mar 22;16(3):474. doi: 10.3390/biom16030474 (PMC13023955; doi:10.3390/biom16030474)
Supplement: Supplementary file 1 [file biomolecules-16-00474-s001.zip › primer sequence.pdf]

Transcript	Primer sequence
mature.tRNA.Arg.CCG_5_end	GACCCAGTGGCCTAATGG
mature.tRNA.Arg.CCT_5_end	GCCCCAGTGGCCTAATGGA
mature.tRNA.Gln.CTG_CCA_end	TCGGTGGAACCTCCA
mature.tRNA.Gln.TTG_CCA_end	CTATTGTCCTAGCCA
mature.tRNA.Ile.AAT	GCCAAGGTCGCGGGTTC
mature.tRNA.Leu.CAA	CAAGTTCTGGTCTCC
mature.tRNA.Leu.CAA_5_end	GTCAGGATGGCCGAGTGGTCTAAGGC
mature.tRNA.Phe.GAA_CCA_end	AATCCCGGGTTTCGGCACCA
mature.tRNA.Ser.CGA_3_end	GGTTCGAATCCTGTTCGTGACG
mature.tRNA.Thr.AGT	GTGGCTTAGCTGGTT
piR.mmu.34076	ATTGATGACTTACAGTCGGC
piR.mmu.49263731	CGTTTCCCGGCCAAT
piR.mmu.49315442	AGTAGCGCAATGGAT
piR.mmu.6790037	AAAGTTTGGAGCTGAGAT
tsRNA.3001b.AsnGTT	CCCACCCAGGGACGCC
tsRNA.3011b.SerTGA	AACCCTGCTCGCTGC
tsRNA.3015b.LeuAAG.LeuTAG	AATCCCACCGCTGCC
tsRNA.3022b.ArgTCG	AATCCCTTCGTGGTT
tsRNA.3031b.LysCTT	TCGAGCCCCACGTTG
tsRNA.3036b.AlaTGC	ATCCCCGGCACCTCC
