# Supplementary material for: Endogenous and Exogenous Small RNA Signatures as Novel Tools for Postmortem Interval Determination
Source: Biomolecules. 2026 Mar 22;16(3):474. doi: 10.3390/biom16030474 (PMC13023955; doi:10.3390/biom16030474)
Supplement: Supplementary file 1 [file biomolecules-16-00474-s001.zip › s3.pdf]

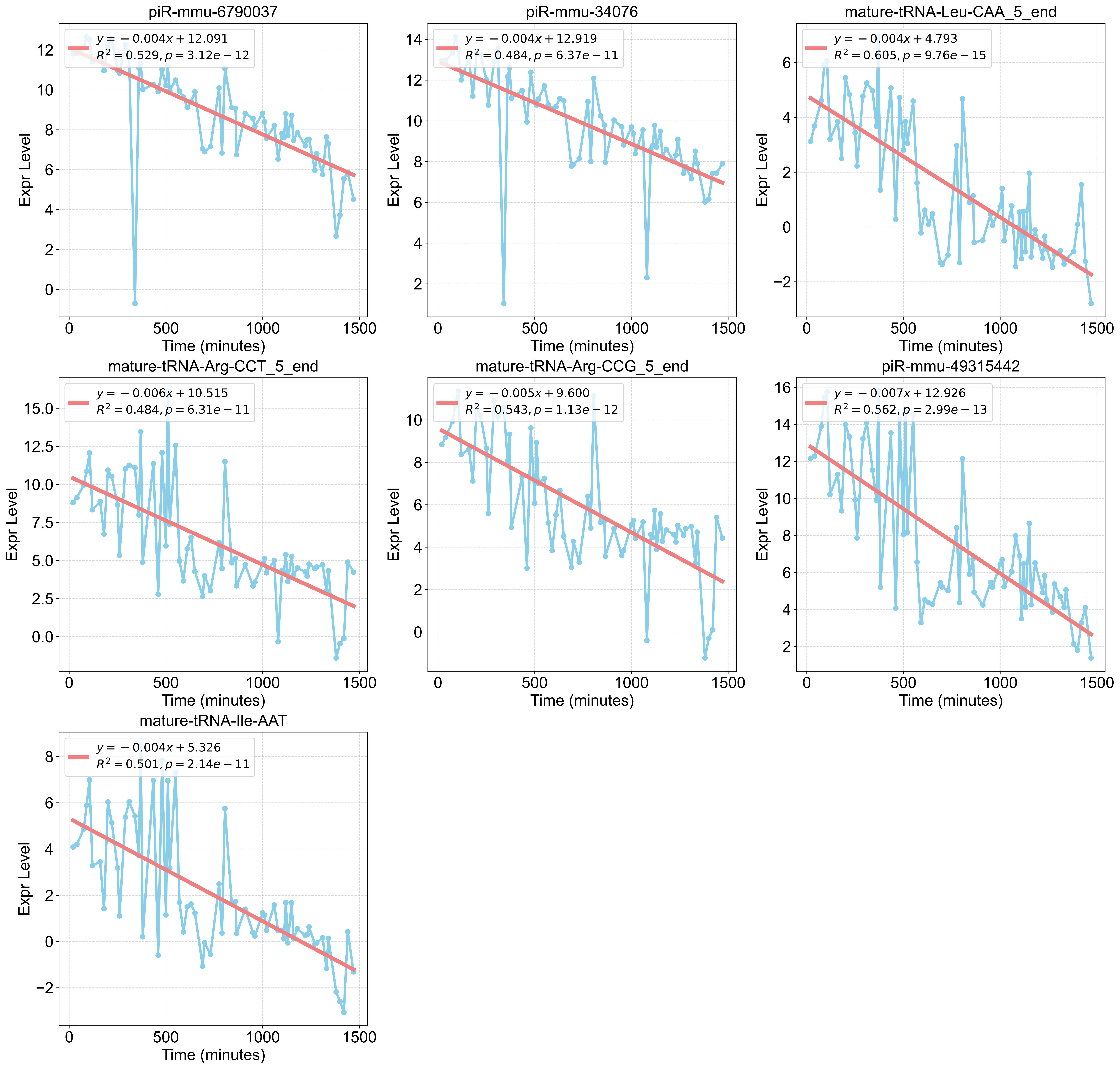

piR-mmu-6790037
$y = -0.004x + 12.091$
$R^2 = 0.529, p = 3.12e-12$
piR-mmu-34076
$y = -0.004x + 12.919$
$R^2 = 0.484, p = 6.37e-11$
mature-tRNA-Leu-CAA_5_end
$y = -0.004x + 4.793$
$R^2 = 0.605, p = 9.76e-15$
mature-tRNA-Arg-CCT_5_end
$y = -0.006x + 10.515$
$R^2 = 0.484, p = 6.31e-11$
mature-tRNA-Arg-CCG_5_end
$y = -0.005x + 9.600$
$R^2 = 0.543, p = 1.13e-12$
piR-mmu-49315442
$y = -0.007x + 12.926$
$R^2 = 0.562, p = 2.99e-13$
mature-tRNA-Ile-AAT
$y = -0.004x + 5.326$
$R^2 = 0.501, p = 2.14e-11$
Expr Level
Time (minutes)
