# Supplementary material for: Endogenous and Exogenous Small RNA Signatures as Novel Tools for Postmortem Interval Determination
Source: Biomolecules. 2026 Mar 22;16(3):474. doi: 10.3390/biom16030474 (PMC13023955; doi:10.3390/biom16030474)

Supplementary Information

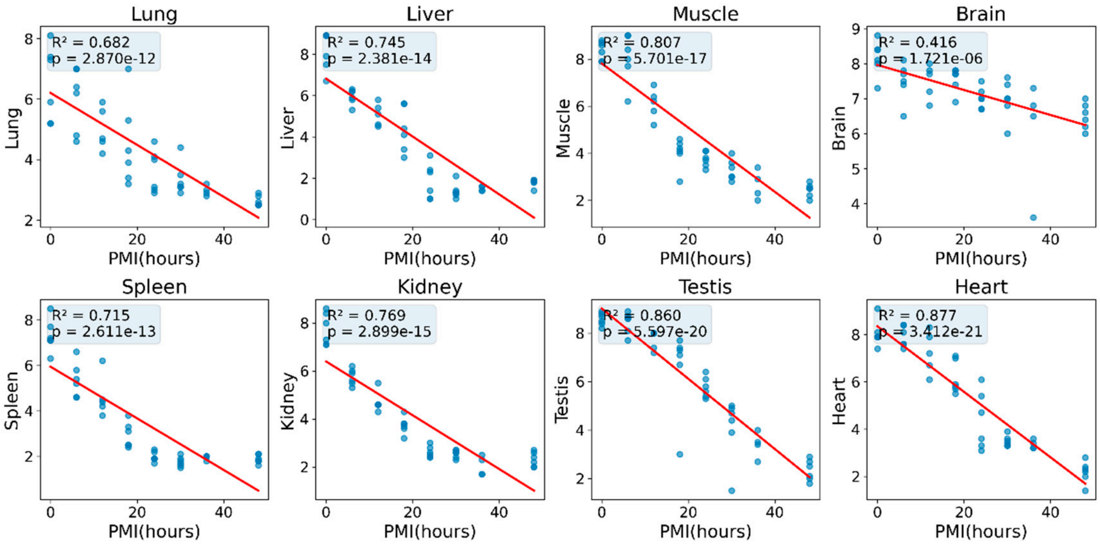

Supplementary Figure S1. Each subpanel displays the distribution of RIN values and their linear fitting results for a specific organ (e.g., heart, liver, brain, etc.) at different postmortem intervals. The red solid line represents the fitted regression line, while the scattered points indicate the raw data points. The annotations show the coefficient of determination (R^2) and the p-value from significance testing, which are used to evaluate the goodness-of-fit and statistical significance of the temporal changes in RIN values for each organ.

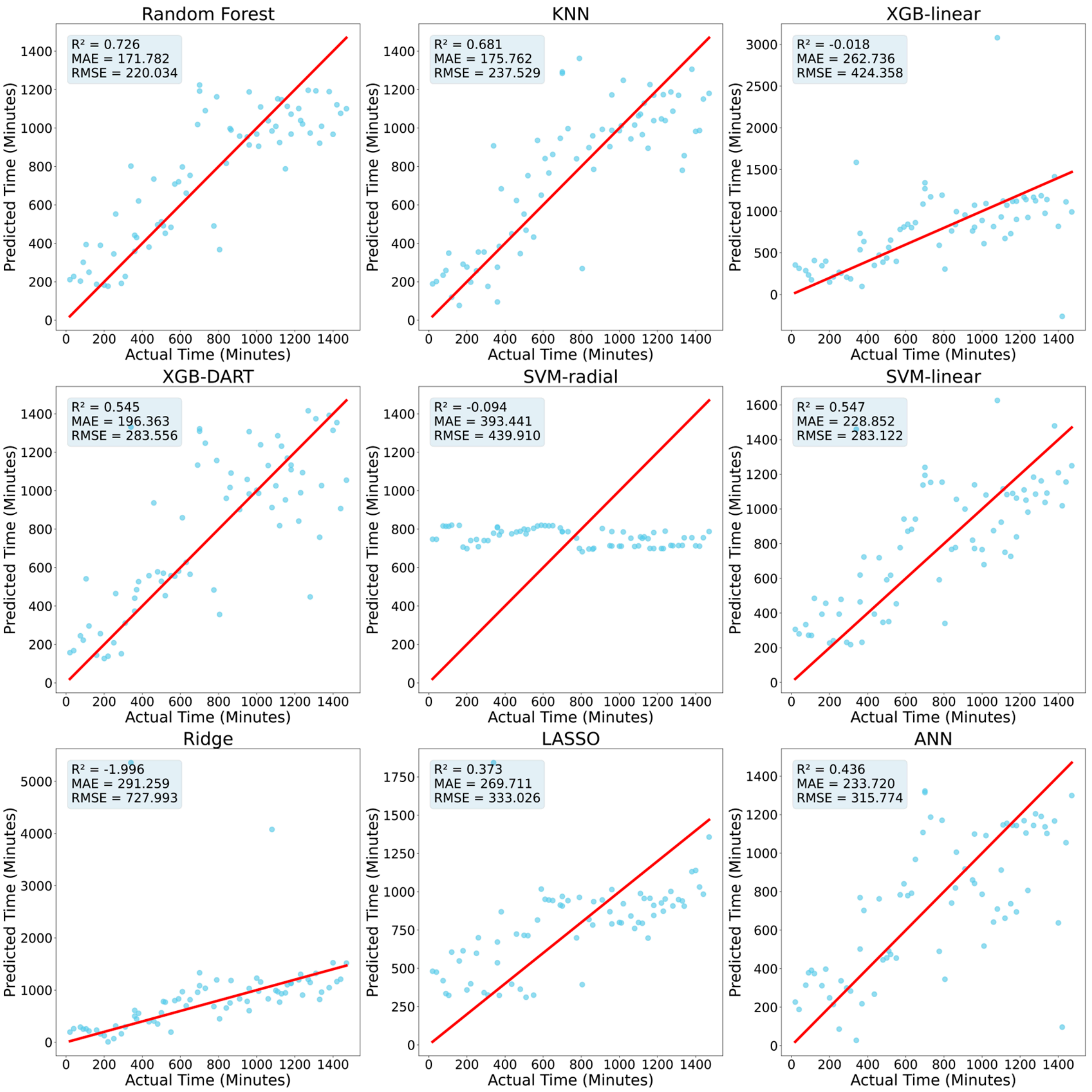

Supplementary Figure S2. Each subpanel presents the performance evaluation of regression models (including RF, KNN, XGB-linear, XGB-DART, SVM-radial, SVM-linear, Ridge, LASSO, and ANN) for PCR array data of 20 sncRNAs. The red diagonal line represents the ideal estimation outcome. Evaluation metrics—R^2, MAE, and RMSE—are displayed in the upper-left corner (with both MAE and RMSE reported in minutes).

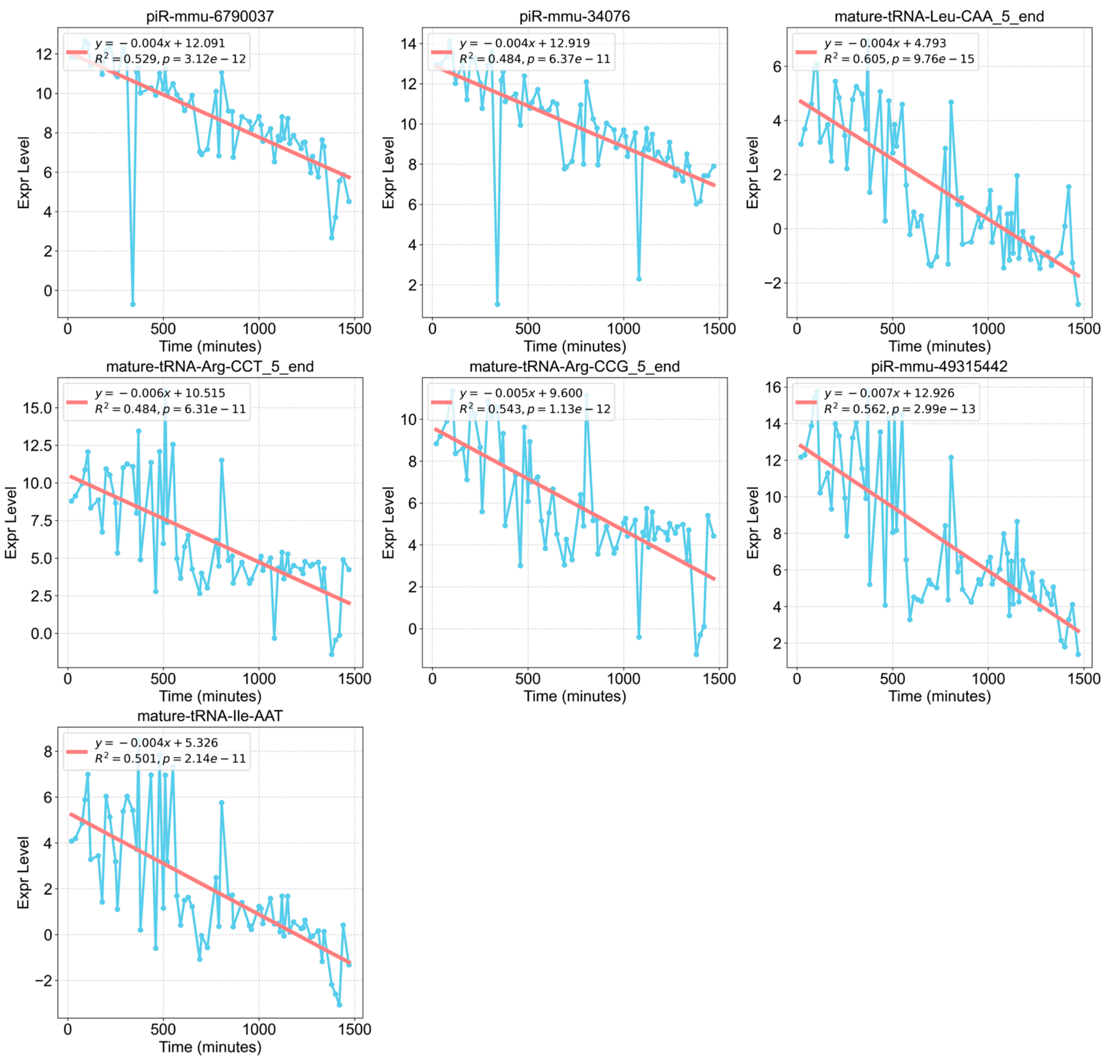

Supplementary Figure S3. Temporal expression patterns and linear fitting of seven biomarkers. Each subplot displays the trend of expression levels over time with corresponding linear regression, where the upper-left corner annotates the linear equation, R^2, and p-value.

Transcript	Primer sequence
mature.tRNA.Arg.CCG_5_end	GACCCAGTGGCCTAATGG
mature.tRNA.Arg.CCT_5_end	GCCCCAGTGGCCTAATGGA
mature.tRNA.Gln.CTG_CCA_end	TCGGTGGAACCTCCA
mature.tRNA.Gln.TTG_CCA_end	CTATTGTCCTAGCCA
mature.tRNA.Ile.AAT	GCCAAGGTCGCGGGTTC
mature.tRNA.Leu.CAA	CAAGTTCTGGTCTCC
mature.tRNA.Leu.CAA_5_end	GTCAGGATGGCCGAGTGGTCTAAGGC
mature.tRNA.Phe.GAA_CCA_end	AATCCCGGGTTTCGGCACCA
mature.tRNA.Ser.CGA_3_end	GGTTCGAATCCTGTTCGTGACG
mature.tRNA.Thr.AGT	GTGGCTTAGCTGGTT
piR.mmu.34076	ATTGATGACTTACAGTCGGC
piR.mmu.49263731	CGTTTCCCGGCCAAT
piR.mmu.49315442	AGTAGCGCAATGGAT
piR.mmu.6790037	AAAGTTTGGAGCTGAGAT
tsRNA.3001b.AsnGTT	CCCACCCAGGGACGCC
tsRNA.3011b.SerTGA	AACCCTGCTCGCTGC
tsRNA.3015b.LeuAAG.LeuTAG	AATCCCACCGCTGCC
tsRNA.3022b.ArgTCG	AATCCCTTCGTGGTT
tsRNA.3031b.LysCTT	TCGAGCCCCACGTTG
tsRNA.3036b.AlaTGC	ATCCCCGGCACCTCC

Supplementary Table S1. Primer sequences for 20 biomarkers

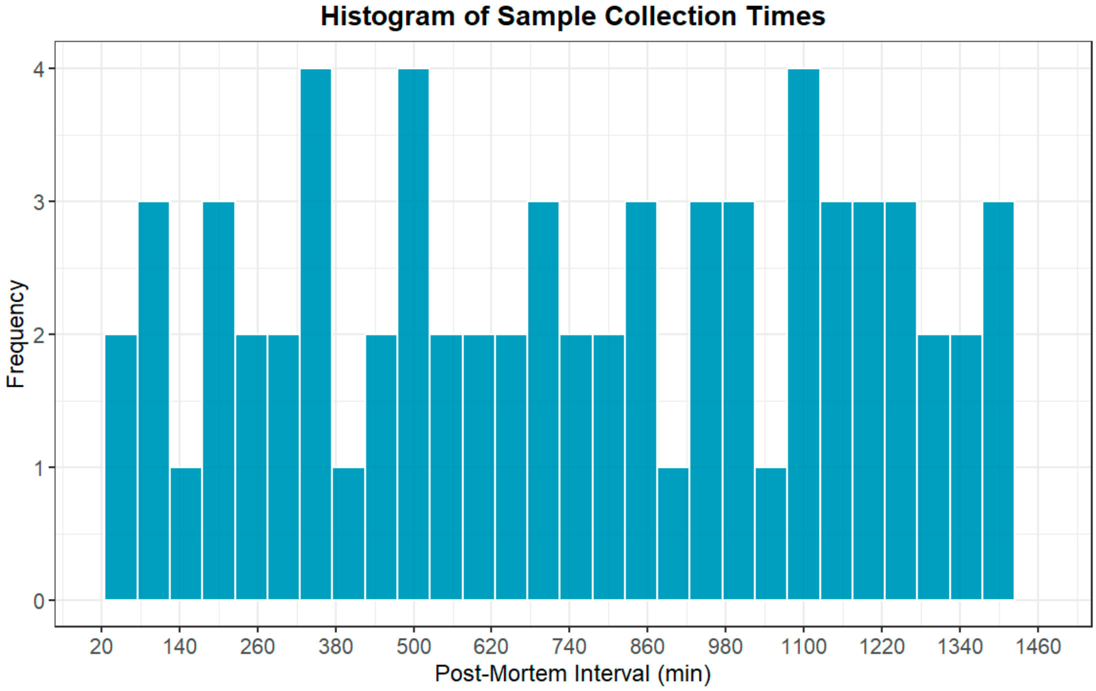

Supplementary Figure S4: histogram of PMI distribution of all samples.

Supplementary Figure S5: PCR Array reaction conditions

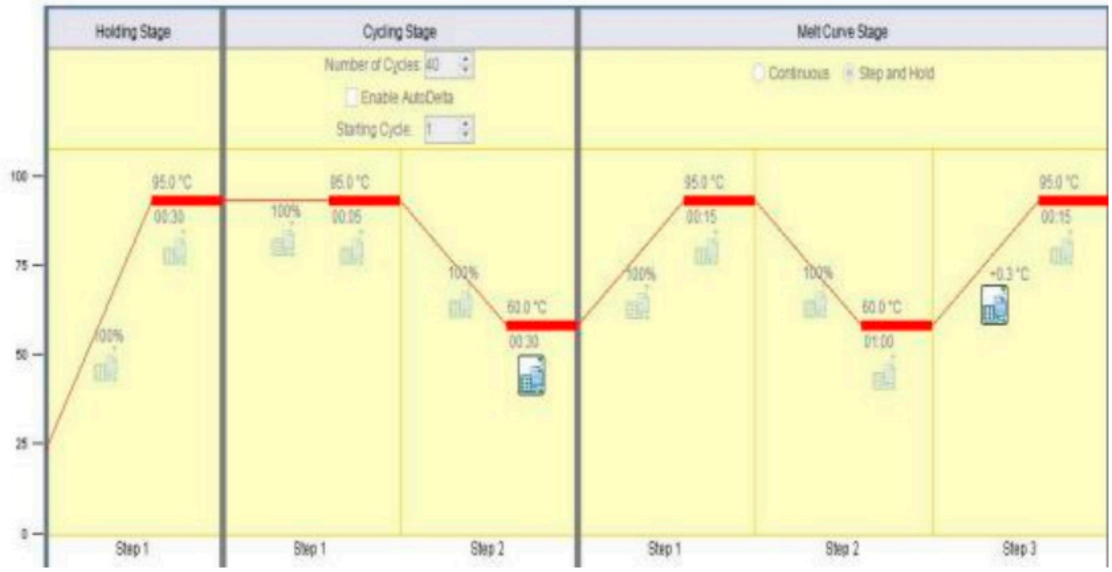

Supplement: Supplementary file 1 [file biomolecules-16-00474-s001.zip › Supplementary Information.pdf]
