# Supplementary figures and images for: Endogenous and Exogenous Small RNA Signatures as Novel Tools for Postmortem Interval Determination
Source: Biomolecules. 2026 Mar 22;16(3):474. doi: 10.3390/biom16030474 (PMC13023955; doi:10.3390/biom16030474)

# Histogram of Sample Collection Times

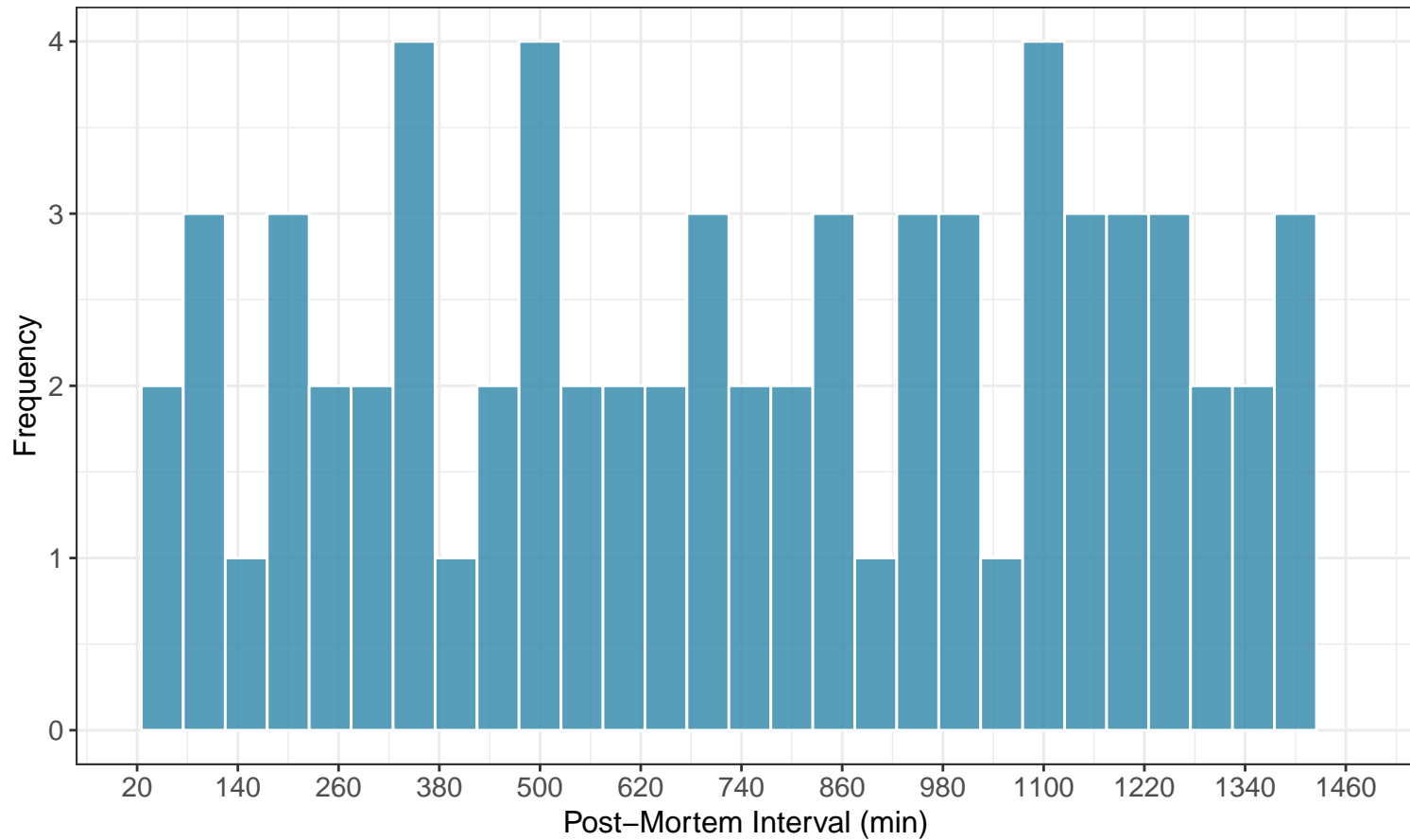

Supplement: Supplementary file 1 [file biomolecules-16-00474-s001.zip › histogram of PMI distribution.pdf]

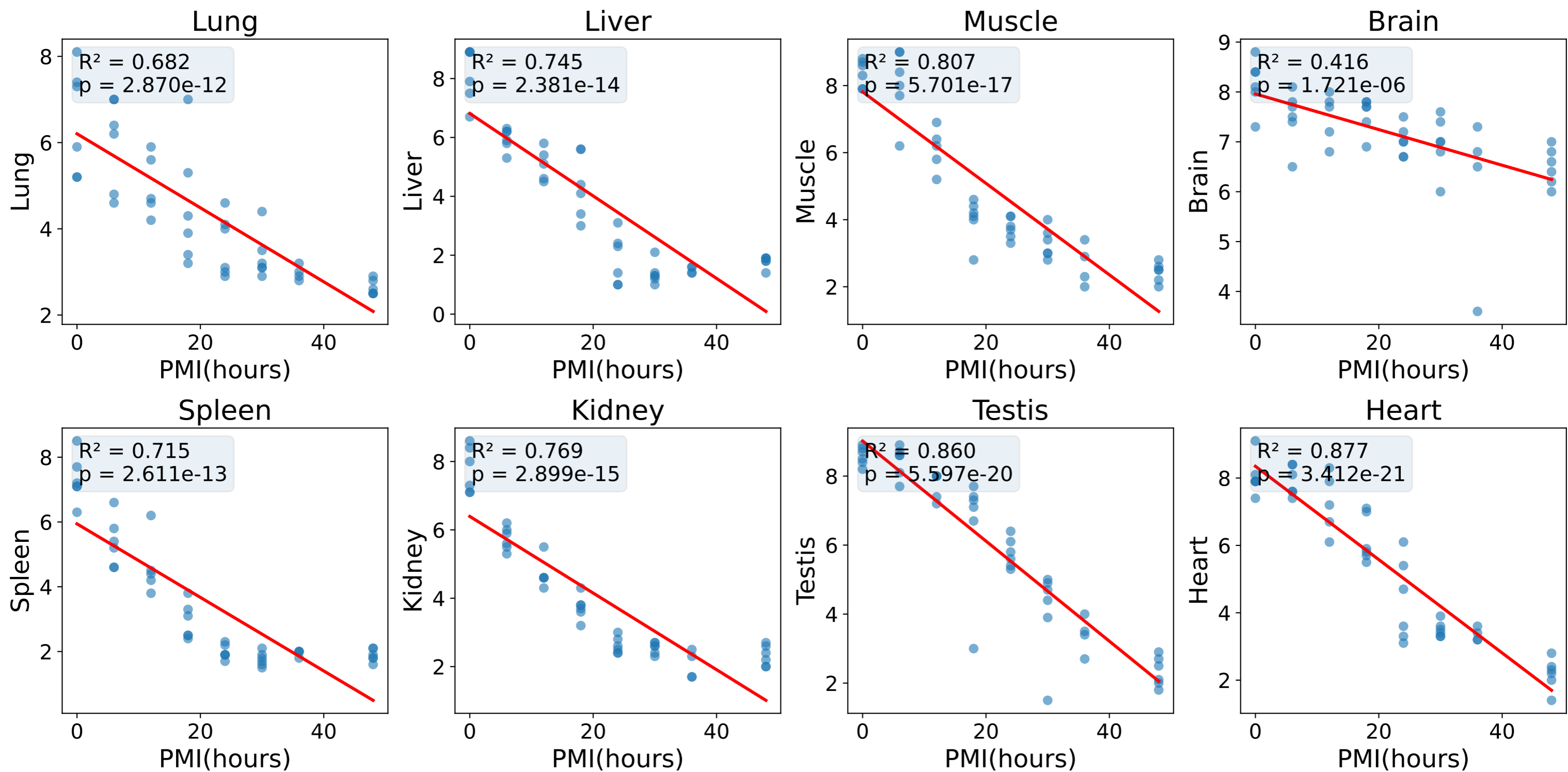

Supplement: Supplementary file 1 [file biomolecules-16-00474-s001.zip › s1.pdf]

Random Forest

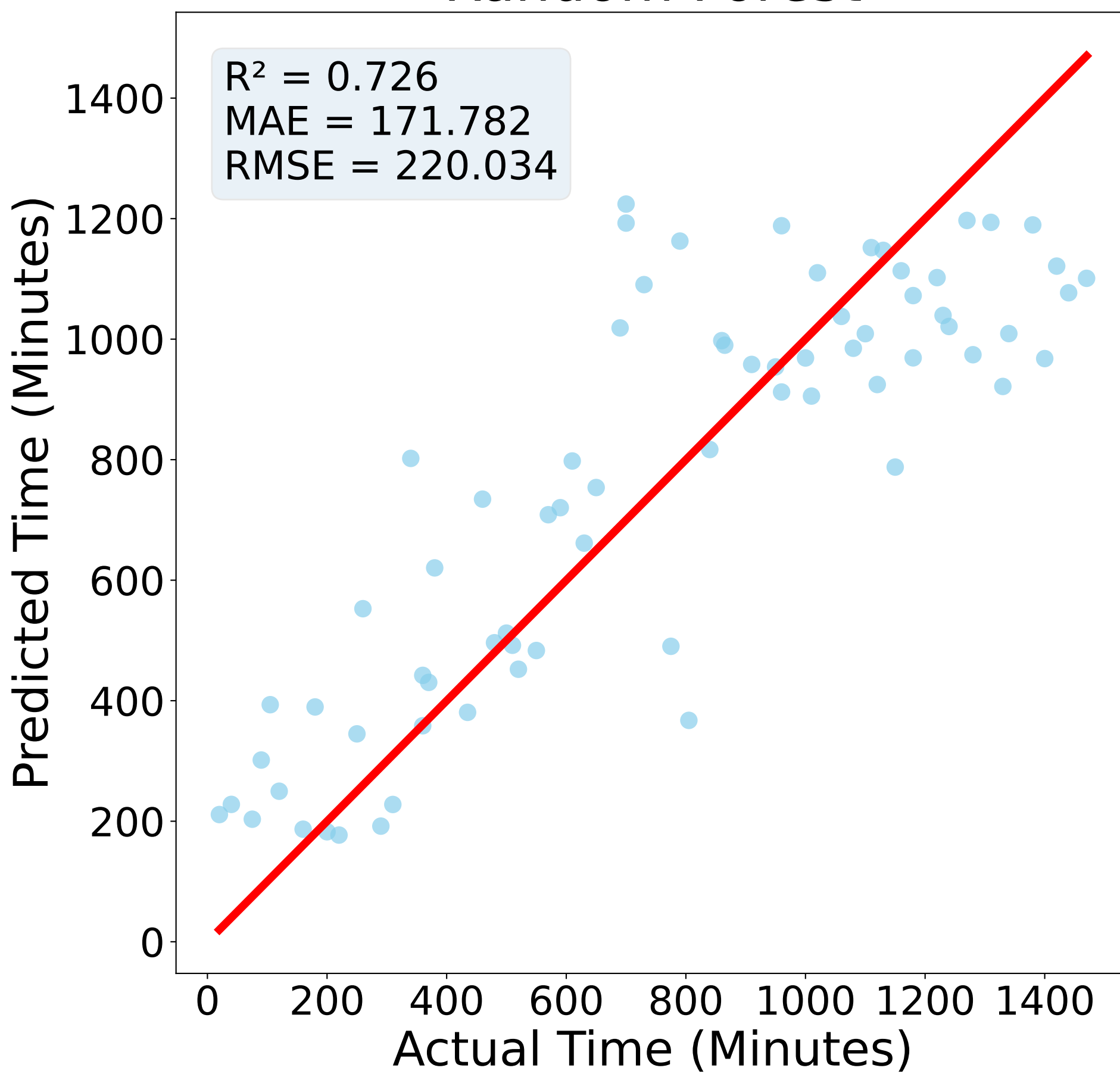

KNN

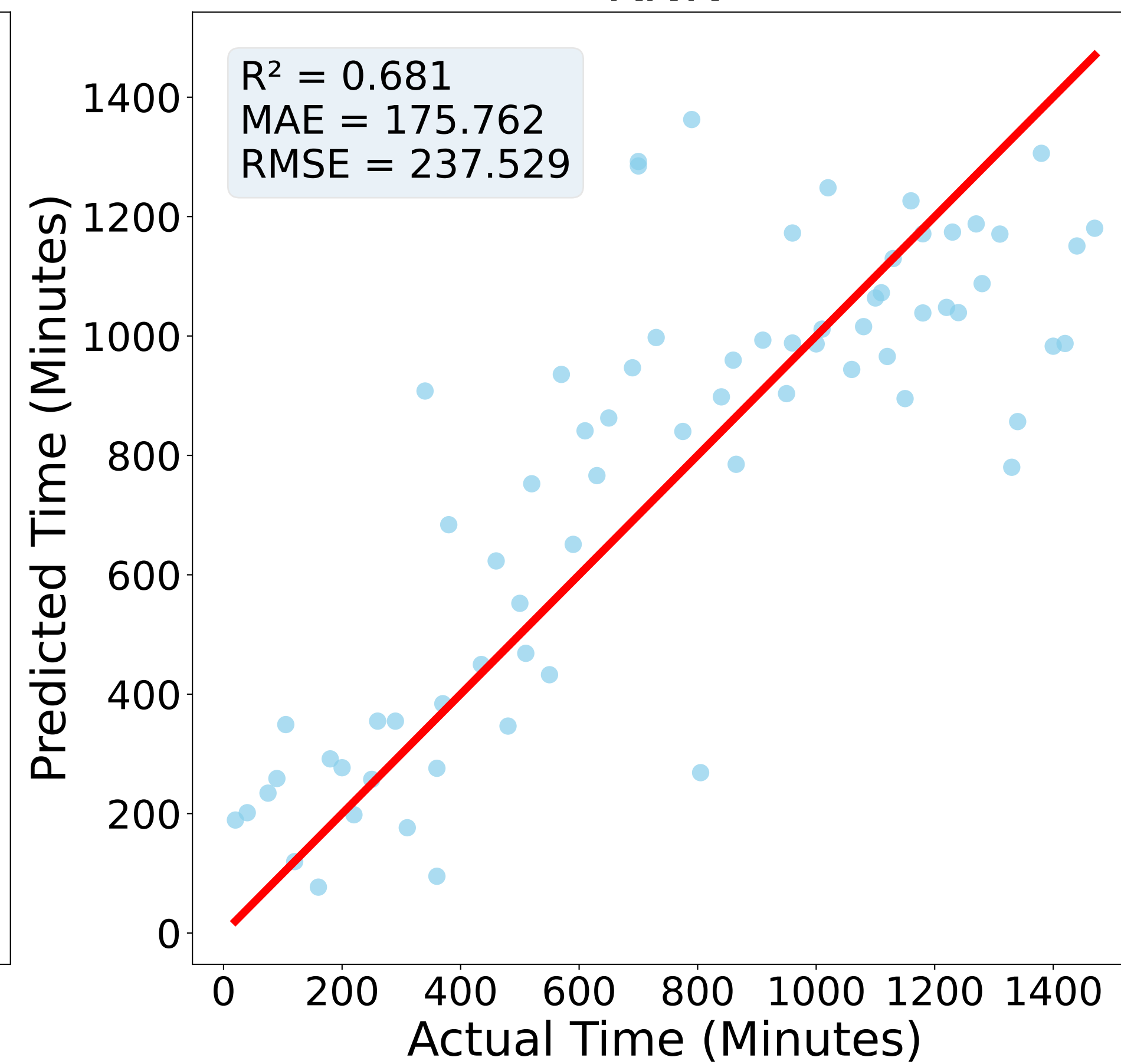

XGB-linear

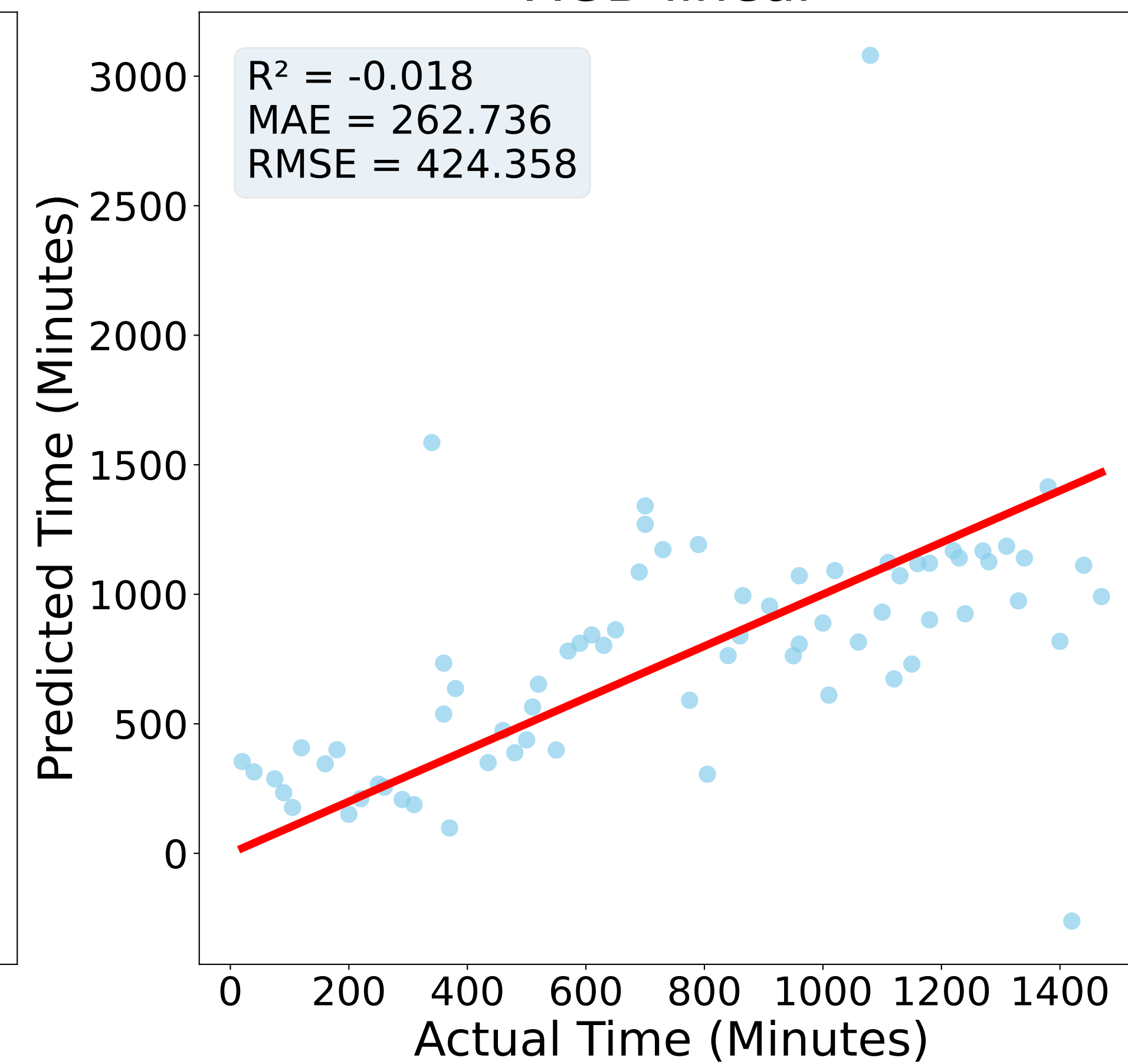

XGB-DART

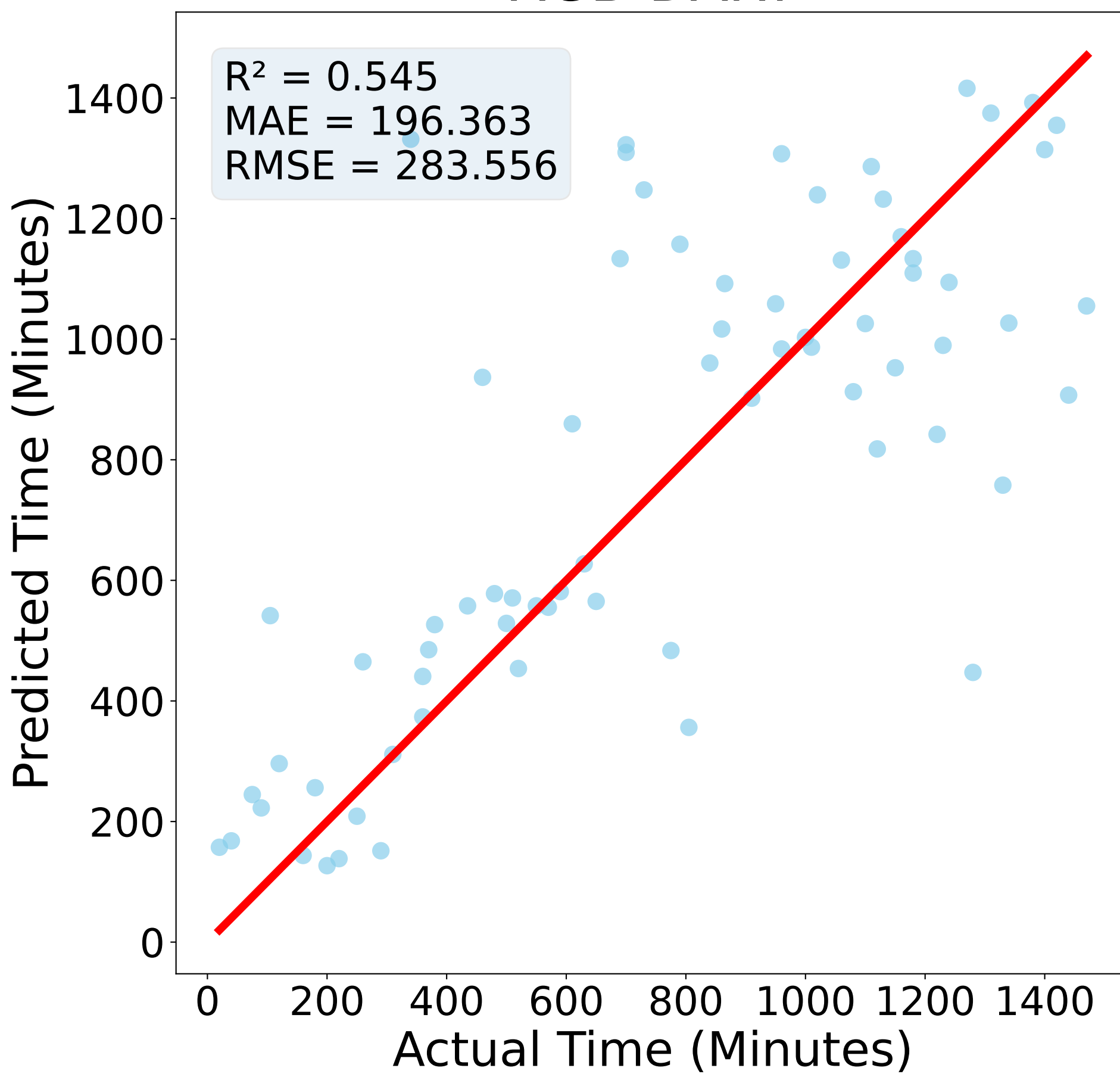

SVM-radial

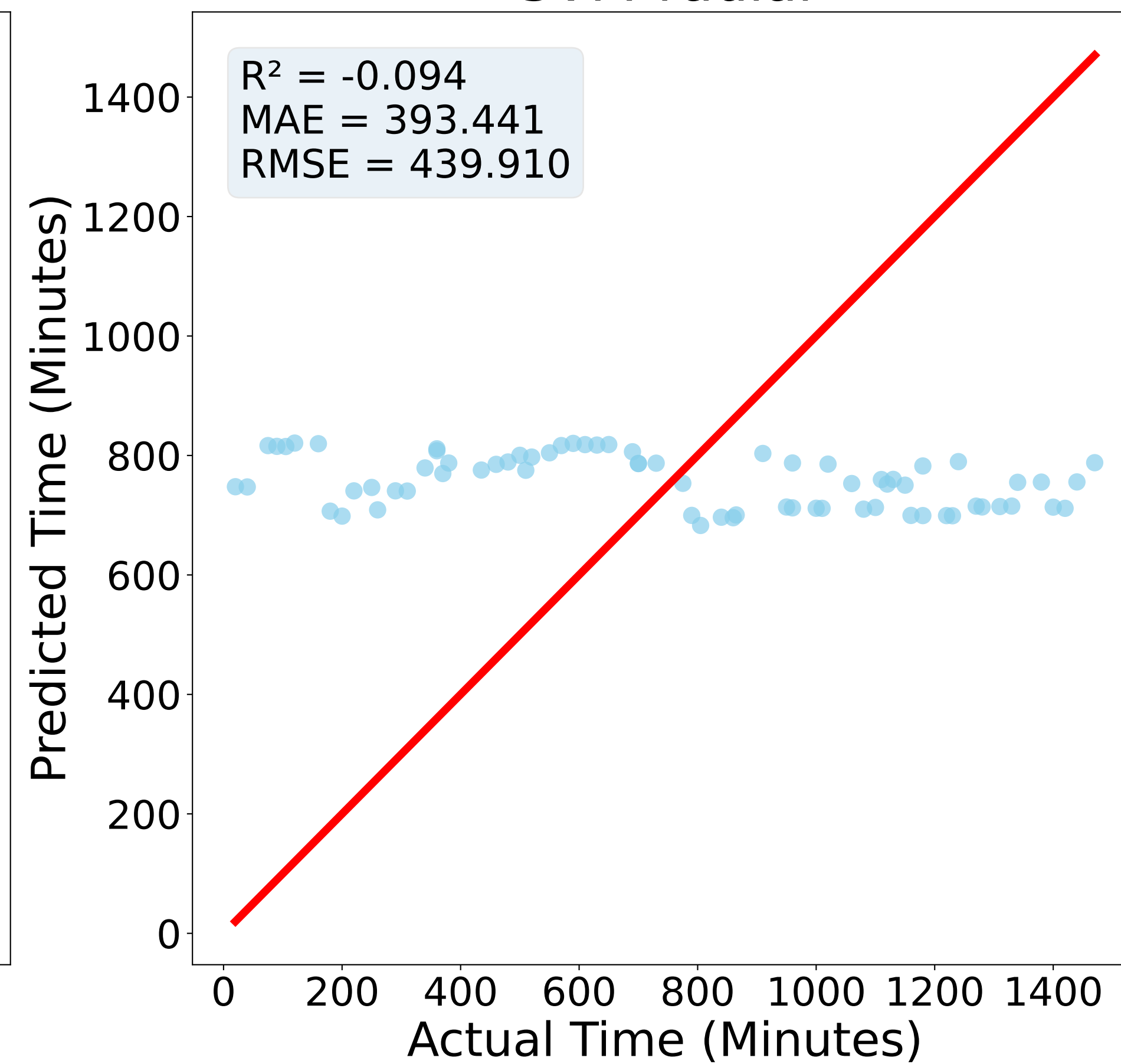

SVM-linear

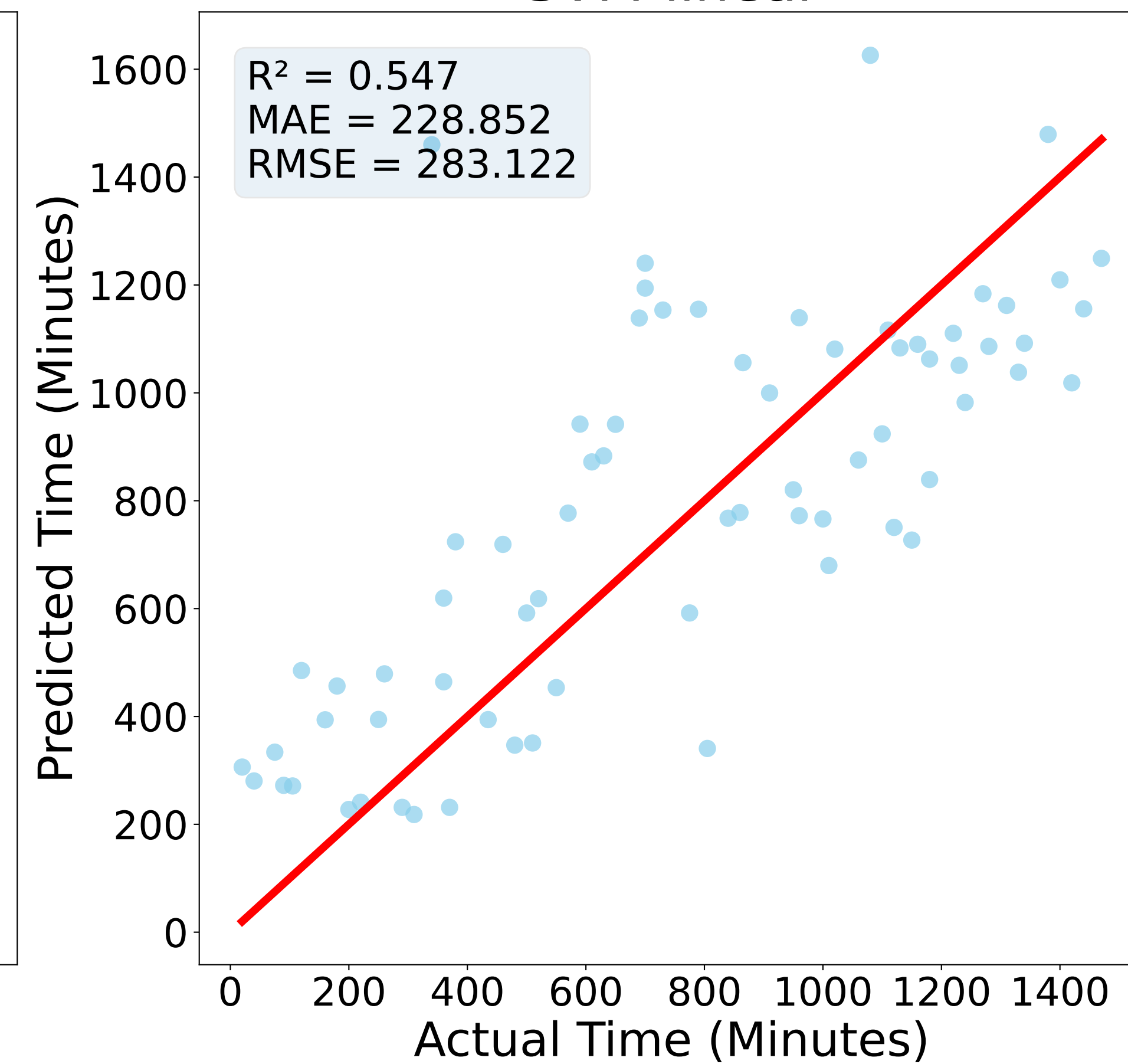

Ridge

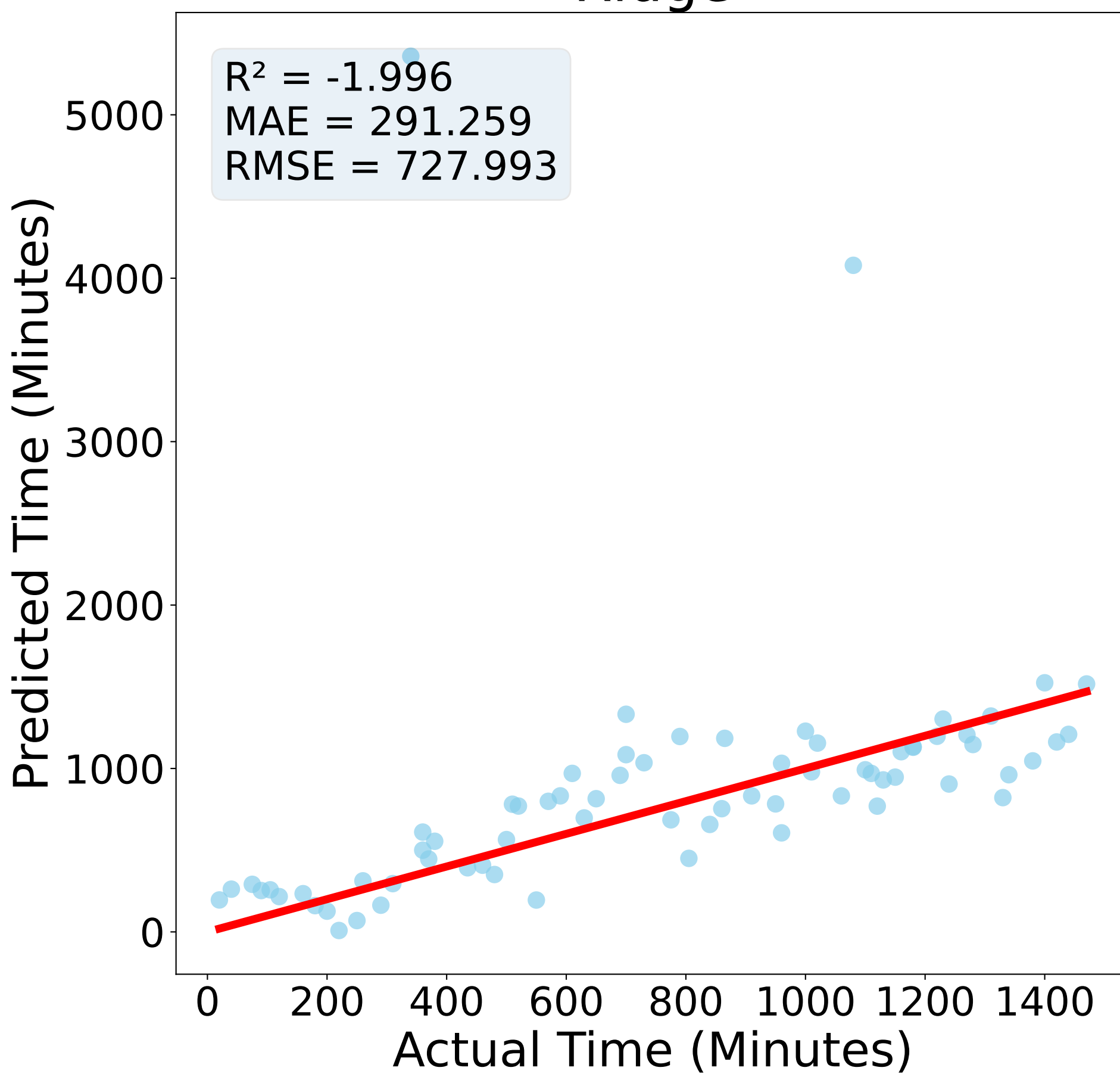

LASSO

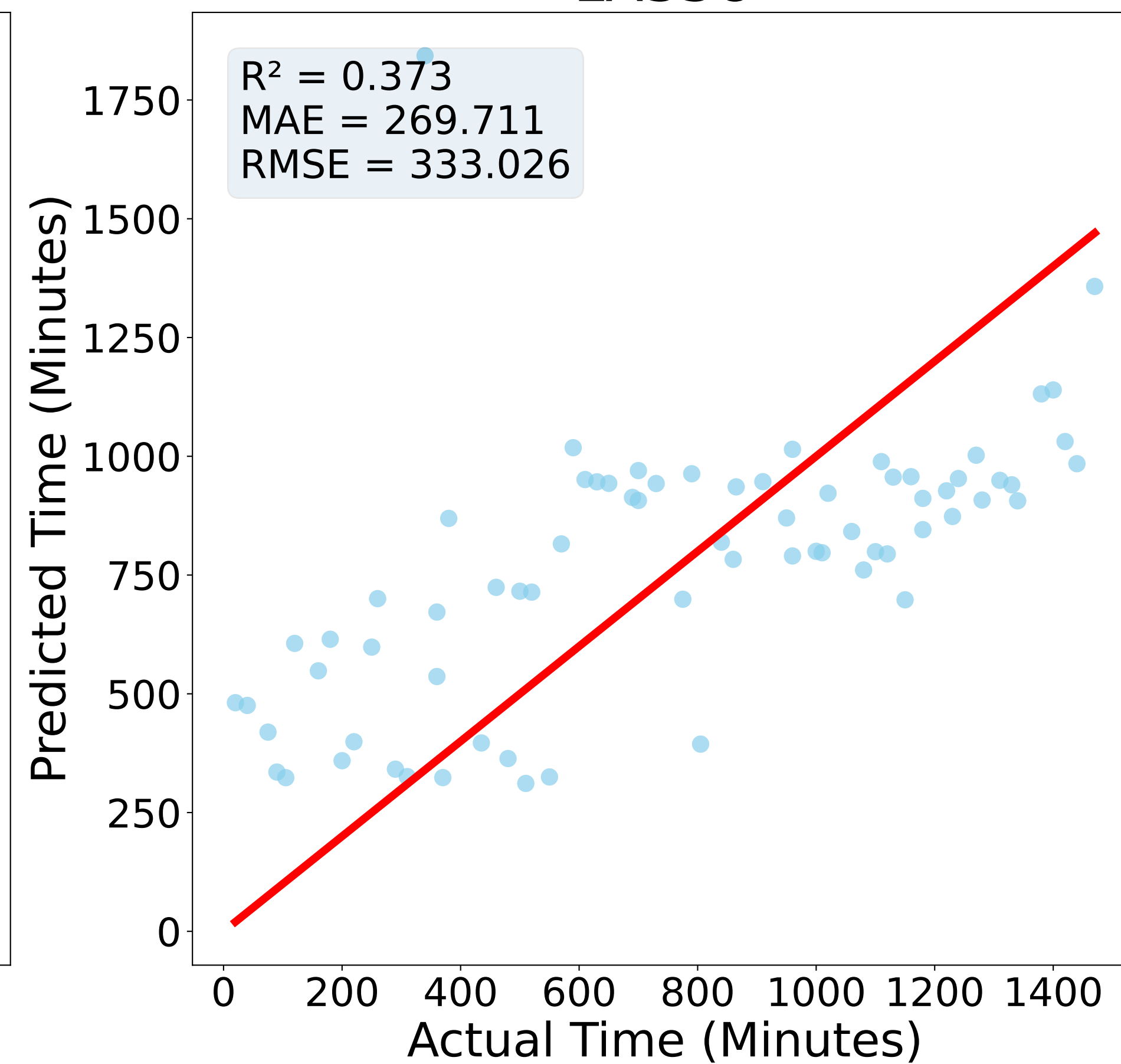

ANN

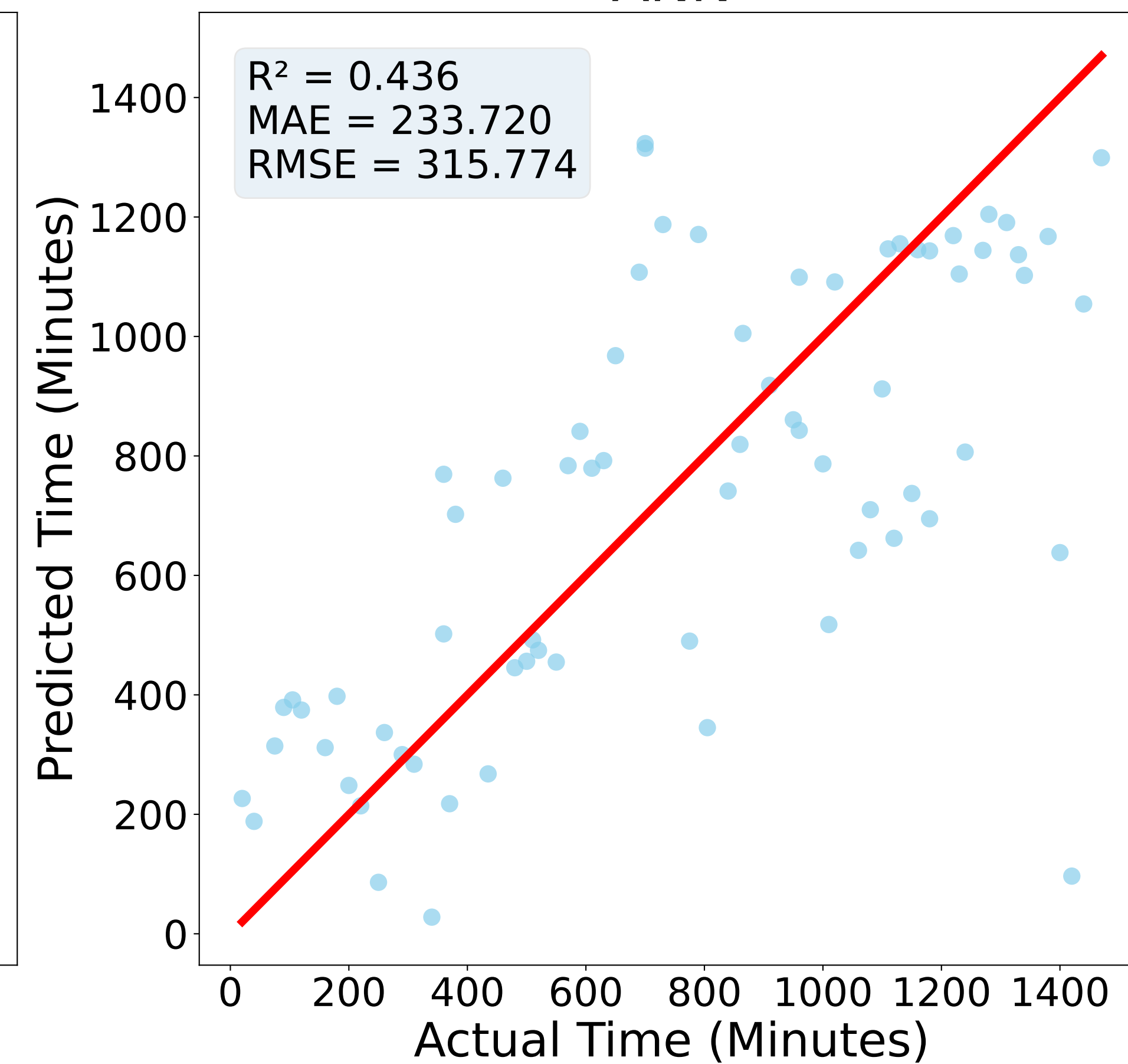

Supplement: Supplementary file 1 [file biomolecules-16-00474-s001.zip › s2.pdf]

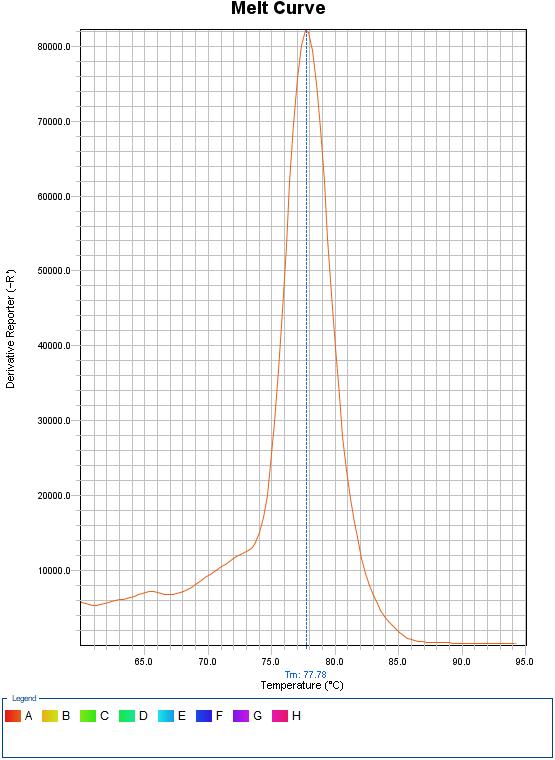

Supplement: Supplementary file 1 [file biomolecules-16-00474-s001.zip › _/Melt Curve-mature.tRNA.Arg.CCG_5_end.jpg]

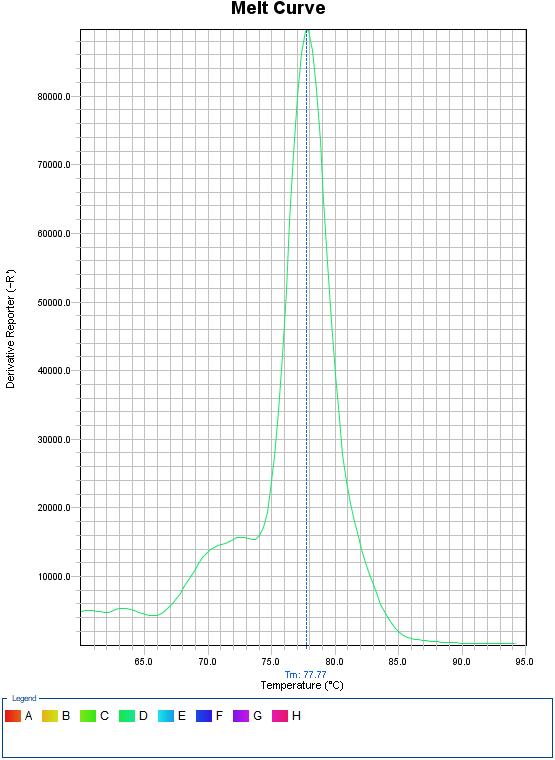

Supplement: Supplementary file 1 [file biomolecules-16-00474-s001.zip › _/Melt Curve-mature.tRNA.Arg.CCT_5_end.jpg]

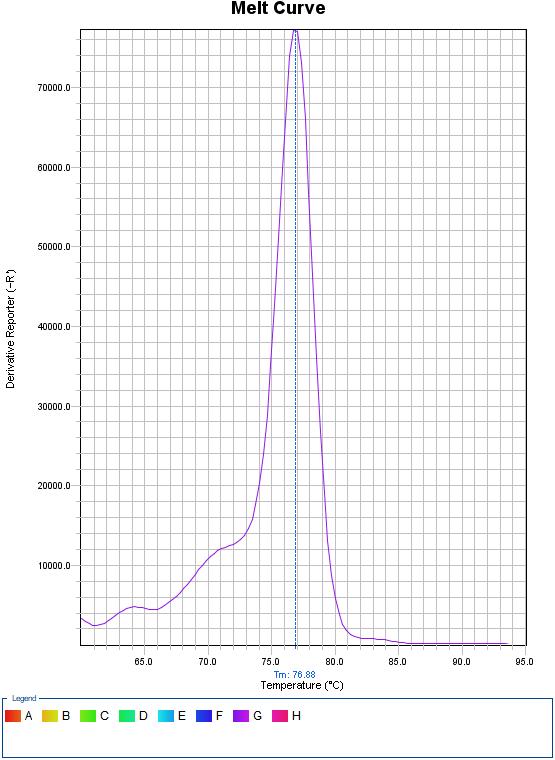

Supplement: Supplementary file 1 [file biomolecules-16-00474-s001.zip › _/Melt Curve-mature.tRNA.Gln.CTG_CCA_end.jpg]

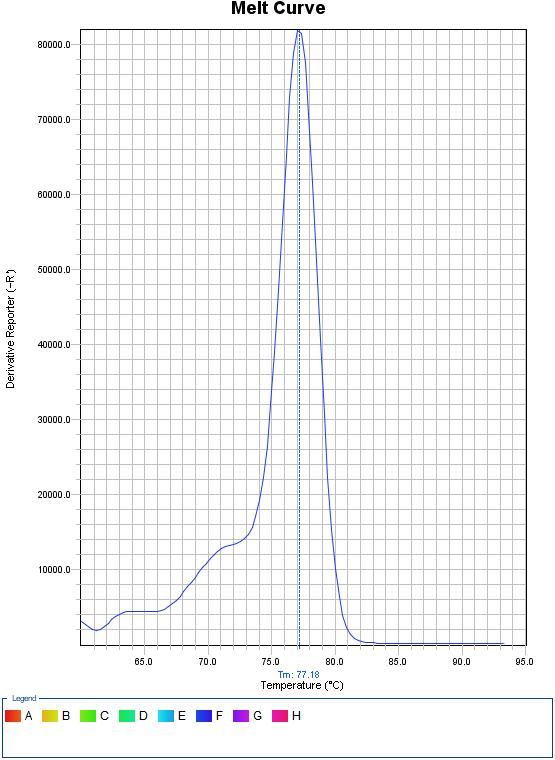

Supplement: Supplementary file 1 [file biomolecules-16-00474-s001.zip › _/Melt Curve-mature.tRNA.Gln.TTG_CCA_end.jpg]

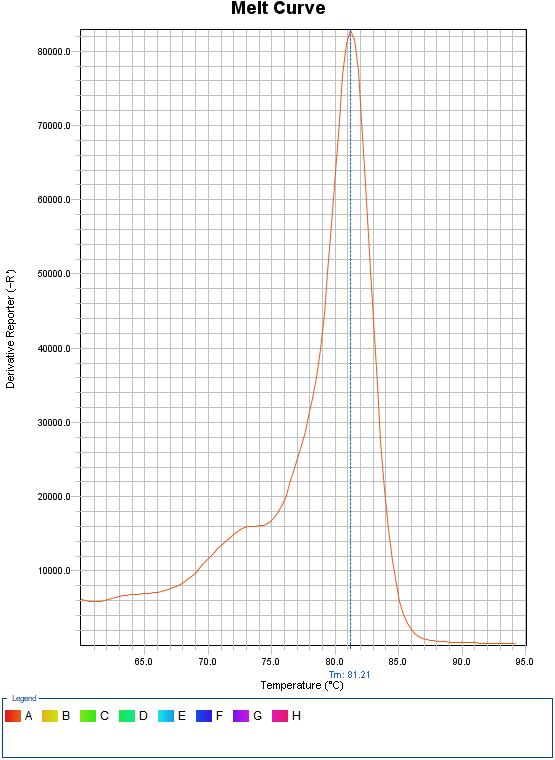

Supplement: Supplementary file 1 [file biomolecules-16-00474-s001.zip › _/Melt Curve-mature.tRNA.Ile.AAT.jpg]

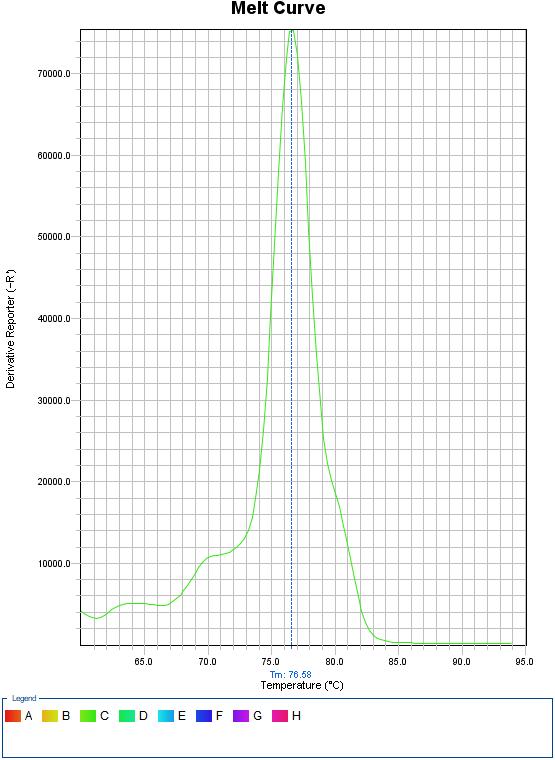

Supplement: Supplementary file 1 [file biomolecules-16-00474-s001.zip › _/Melt Curve-mature.tRNA.Leu.CAA.jpg]

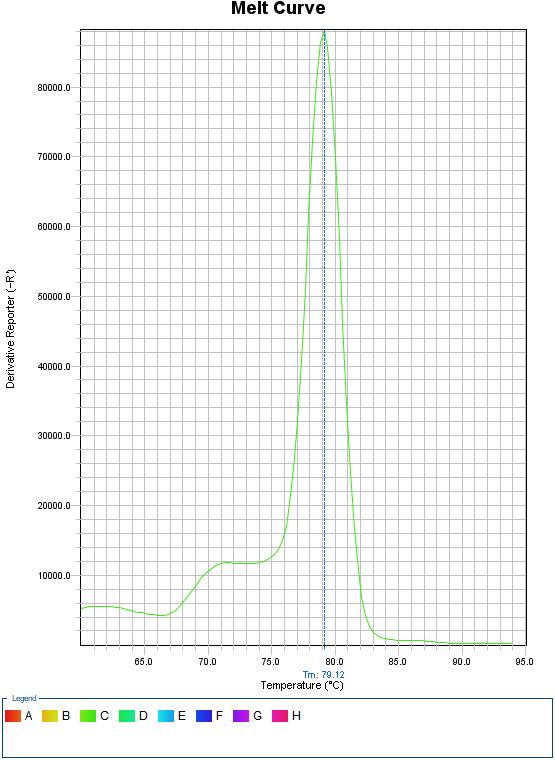

Supplement: Supplementary file 1 [file biomolecules-16-00474-s001.zip › _/Melt Curve-mature.tRNA.Leu.CAA_5_end.jpg]

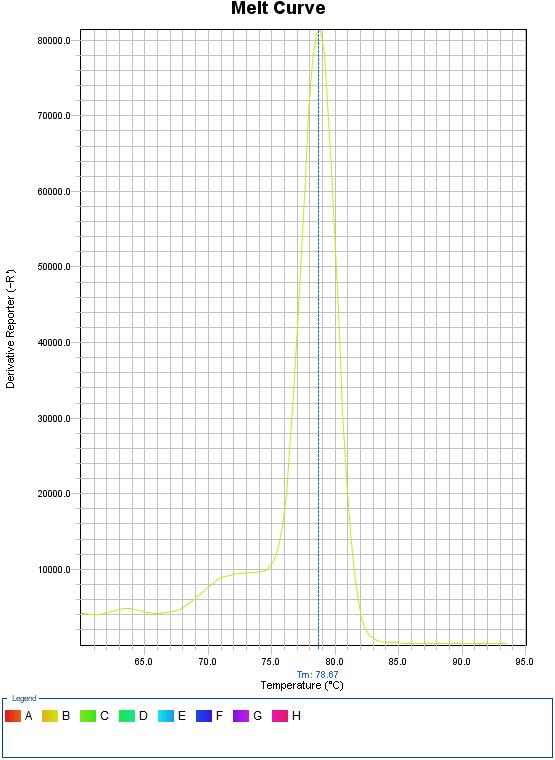

Supplement: Supplementary file 1 [file biomolecules-16-00474-s001.zip › _/Melt Curve-mature.tRNA.Phe.GAA_CCA_end.jpg]

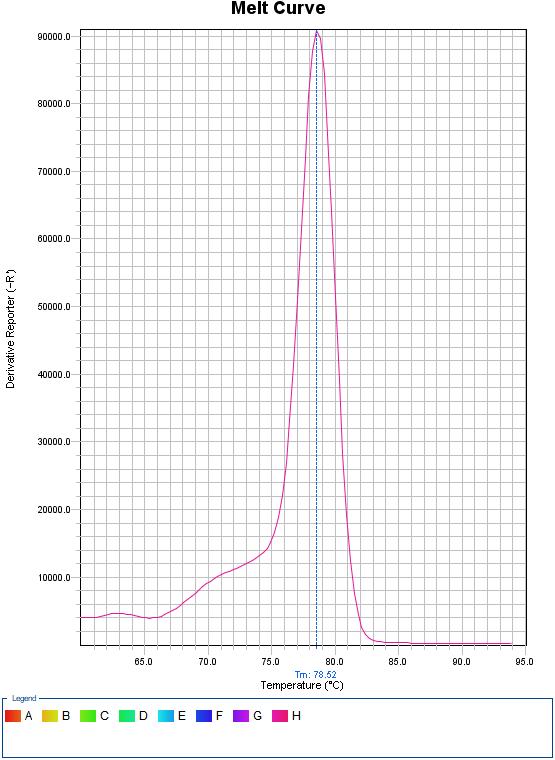

Supplement: Supplementary file 1 [file biomolecules-16-00474-s001.zip › _/Melt Curve-mature.tRNA.Ser.CGA_3_end.jpg]

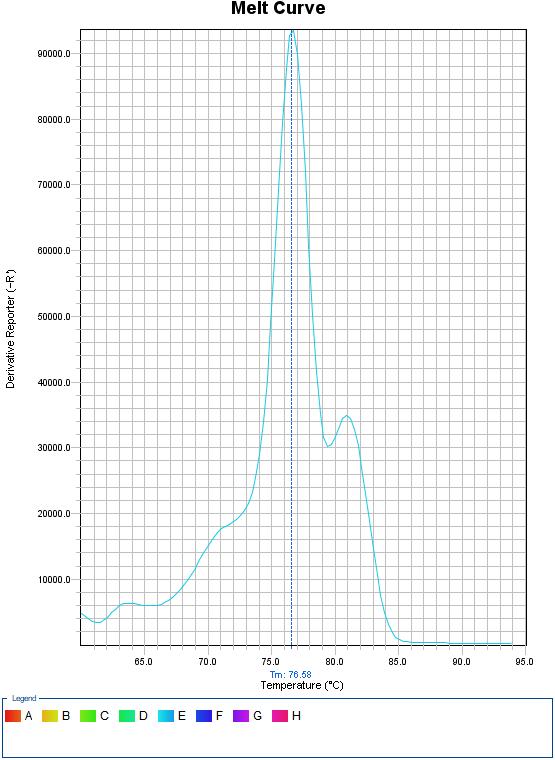

Supplement: Supplementary file 1 [file biomolecules-16-00474-s001.zip › _/Melt Curve-mature.tRNA.Thr.AGT.jpg]

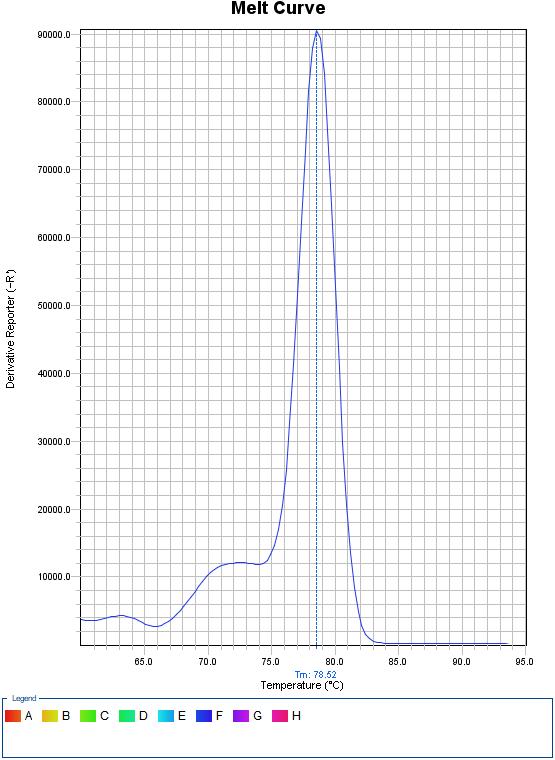

Supplement: Supplementary file 1 [file biomolecules-16-00474-s001.zip › _/Melt Curve-piR.mmu.49263731.jpg]

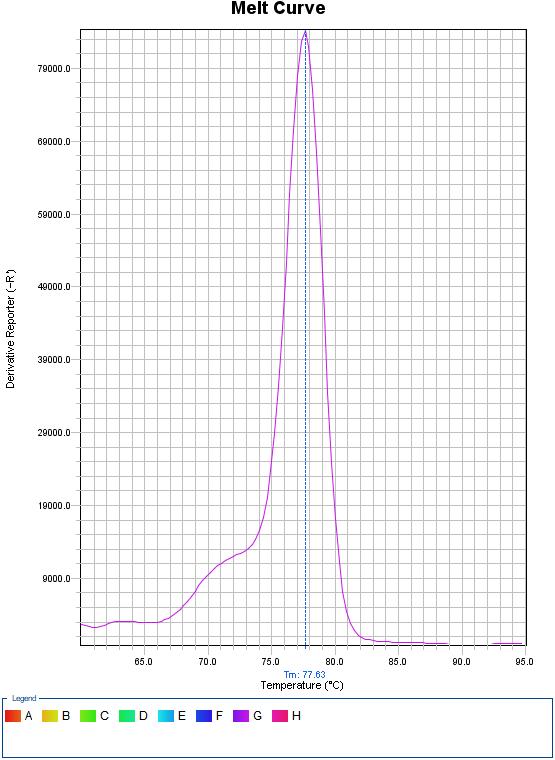

Supplement: Supplementary file 1 [file biomolecules-16-00474-s001.zip › _/Melt Curve-piR.mmu.49315442.jpg]

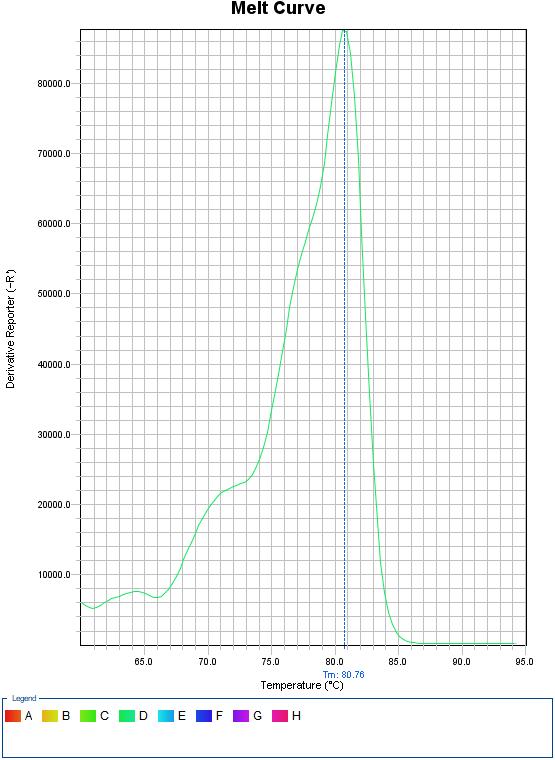

Supplement: Supplementary file 1 [file biomolecules-16-00474-s001.zip › _/Melt Curve-piR.mmu.6790037.jpg]

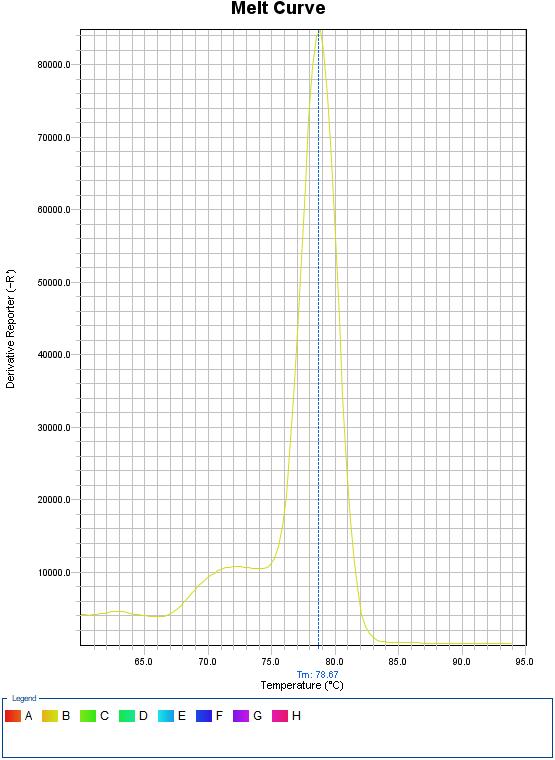

Supplement: Supplementary file 1 [file biomolecules-16-00474-s001.zip › _/Melt Curve-tsRNA.3001b.AsnGTT.jpg]

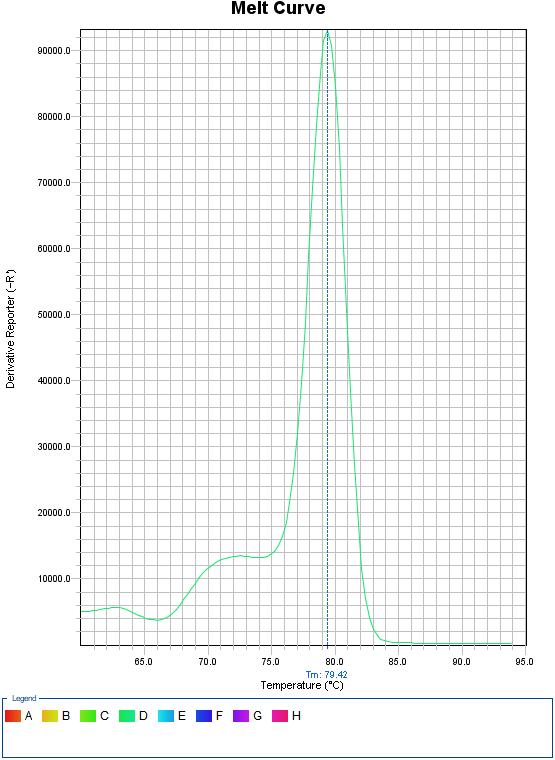

Supplement: Supplementary file 1 [file biomolecules-16-00474-s001.zip › _/Melt Curve-tsRNA.3011b.SerTGA.jpg]

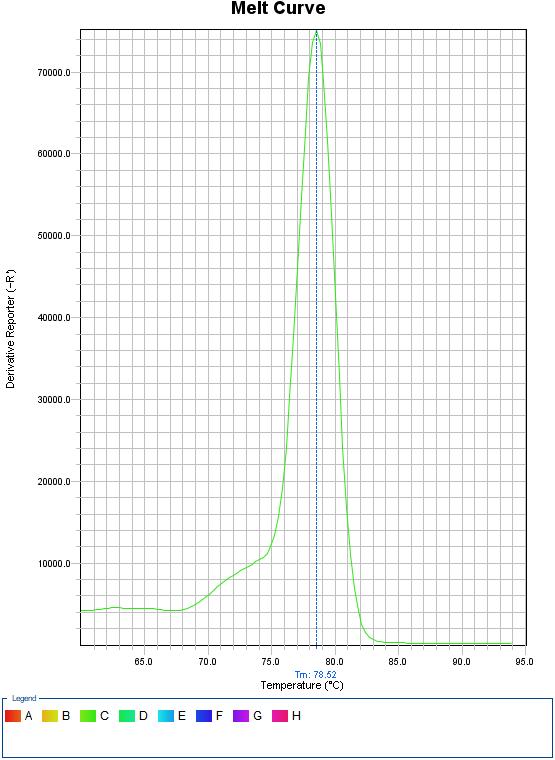

Supplement: Supplementary file 1 [file biomolecules-16-00474-s001.zip › _/Melt Curve-tsRNA.3015b.LeuAAG.LeuTAG.jpg]

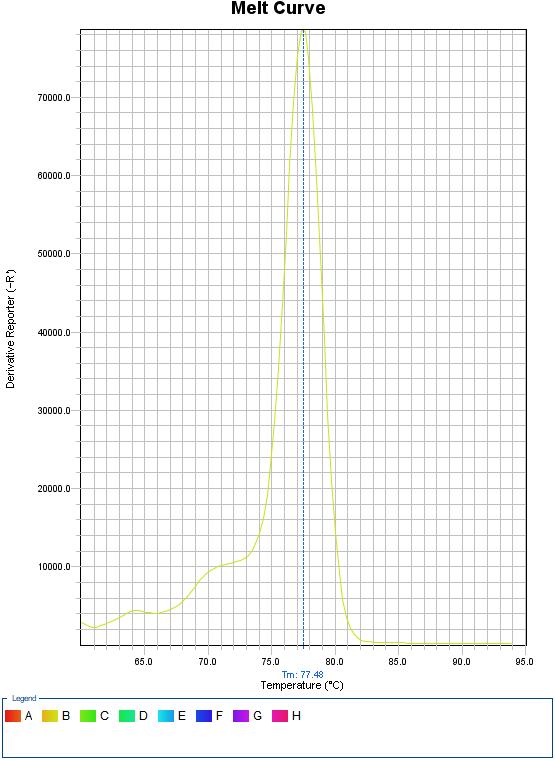

Supplement: Supplementary file 1 [file biomolecules-16-00474-s001.zip › _/Melt Curve-tsRNA.3022b.ArgTCG.jpg]

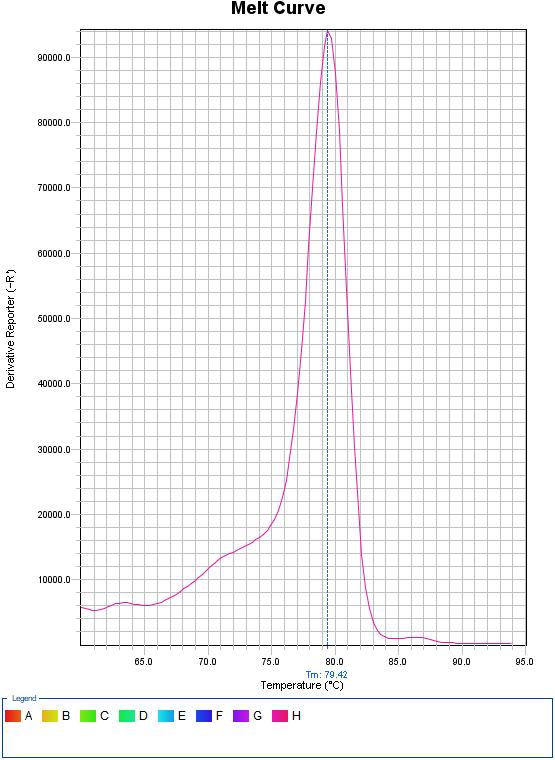

Supplement: Supplementary file 1 [file biomolecules-16-00474-s001.zip › _/Melt Curve-tsRNA.3031b.LysCTT.jpg]

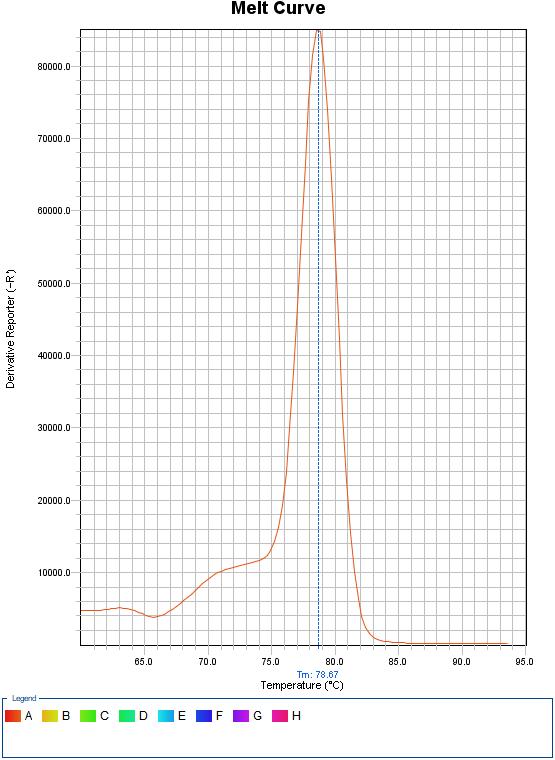

Supplement: Supplementary file 1 [file biomolecules-16-00474-s001.zip › _/Melt Curve-tsRNA.3036b.AlaTGC.jpg]

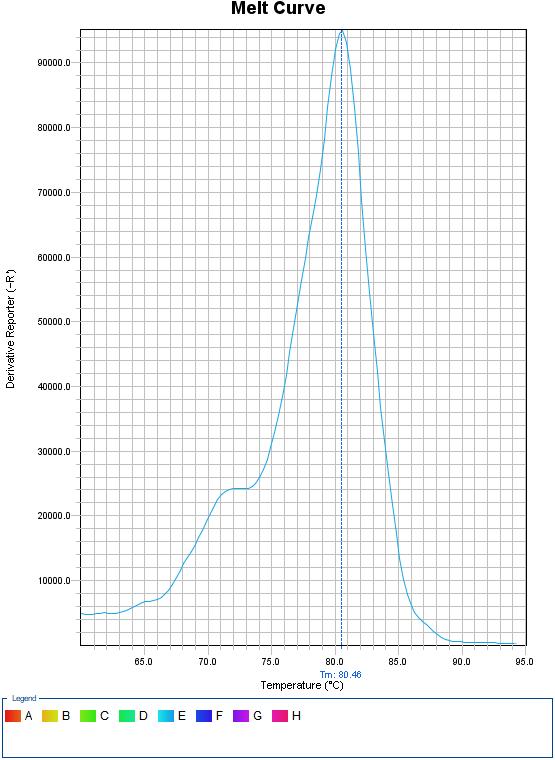

Supplement: Supplementary file 1 [file biomolecules-16-00474-s001.zip › _/Melt CurvepiR.mmu.34076.jpg]
